# A Novel AMPK Inhibitor Sensitizes Pancreatic Cancer Cells to Ferroptosis Induction

**DOI:** 10.1002/advs.202307695

**Published:** 2024-06-17

**Authors:** Carolin Schneider, Jorina Hilbert, Franziska Genevaux, Stefanie Höfer, Lukas Krauß, Felix Schicktanz, Constanza Tapia Contreras, Shaishavi Jansari, Aristeidis Papargyriou, Thorsten Richter, Abdallah M. Alfayomy, Chiara Falcomatà, Christian Schneeweis, Felix Orben, Ruppert Öllinger, Florian Wegwitz, Angela Boshnakovska, Peter Rehling, Denise Müller, Philipp Ströbel, Volker Ellenrieder, Lena Conradi, Elisabeth Hessmann, Michael Ghadimi, Marian Grade, Matthias Wirth, Katja Steiger, Roland Rad, Bernhard Kuster, Wolfgang Sippl, Maximilian Reichert, Dieter Saur, Günter Schneider

**Affiliations:** ^1^ Department of General, Visceral and Pediatric Surgery University Medical Center Göttingen 37075 Göttingen Germany; ^2^ Medical Clinic and Polyclinic II Klinikum rechts der Isar Technical University of Munich 81675 Munich Germany; ^3^ Proteomics and Bioanalytics Department of Molecular Life Sciences School of Life Sciences Technical University of Munich 85354 Freising Germany; ^4^ Institute of Pathology Technical University of Munich 81675 Munich Germany; ^5^ Department of Gynecology and Obstetrics University Medical Center Göttingen Göttingen Germany; ^6^ Institute of Stem Cell Research Helmholtz Zentrum Muenchen D‐85764 Neuherberg Germany; ^7^ Translational Pancreatic Research Cancer Center Medical Clinic and Polyclinic II Klinikum rechts der Isar Technical University of Munich 81675 Munich Germany; ^8^ Center for Organoid Systems (COS) Technical University of Munich 85747 Garching Germany; ^9^ Department of Medicinal Chemistry Institute of Pharmacy Martin‐Luther University Halle‐Wittenberg 06120 Halle (Saale) Germany; ^10^ Department of Pharmaceutical Chemistry Al‐Azhar University Assiut 71524 Egypt; ^11^ Institute for Translational Cancer Research and Experimental Cancer Therapy Technical University Munich 81675 Munich Germany; ^12^ Precision Immunology Institute Icahn School of Medicine at Mount Sinai New York NY USA; ^13^ Institute of Molecular Oncology and Functional Genomics TUM School of Medicine Technical University of Munich 81675 Munich Germany; ^14^ Department of Cellular Biochemistry University Medical Center 37073 Göttingen Germany; ^15^ Max Planck Institute for Biophysical Chemistry 37077 Göttingen Germany; ^16^ Institute of Pathology University Medical Center 37075 Göttingen Germany; ^17^ Clinical Research Unit 5002 KFO5002 University Medical Center Göttingen 37075 Göttingen Germany; ^18^ CCC‐N (Comprehensive Cancer Center Lower Saxony) 37075 Göttingen Germany; ^19^ Department of Gastroenterology Gastrointestinal Oncology and Endocrinology University Medical Center Göttingen 37075 Göttingen Germany; ^20^ Department of Hematology Oncology and Cancer Immunology Campus Benjamin Franklin Charité – Universitätsmedizin Berlin Corporate Member of Freie Universität Berlin and Humboldt‐Universität zu Berlin 12203 Berlin Germany; ^21^ German Cancer Consortium (DKTK) partner site Munich a partnership between DKFZ and University Hospital Klinikum rechts der Isar 81675 München Germany; ^22^ Center for Protein Assemblies (CPA) Technical University of Munich 85747 Garching Germany

**Keywords:** AMPK, ferroptosis, pancreatic cancer

## Abstract

Cancer cells must develop strategies to adapt to the dynamically changing stresses caused by intrinsic or extrinsic processes, or therapeutic agents. Metabolic adaptability is crucial to mitigate such challenges. Considering metabolism as a central node of adaptability, it is focused on an energy sensor, the AMP‐activated protein kinase (AMPK). In a subtype of pancreatic ductal adenocarcinoma (PDAC) elevated AMPK expression and phosphorylation is identified. Using drug repurposing that combined screening experiments and chemoproteomic affinity profiling, it is identified and characterized PF‐3758309, initially developed as an inhibitor of PAK4, as an AMPK inhibitor. PF‐3758309 shows activity in pre‐clinical PDAC models, including primary patient‐derived organoids. Genetic loss‐of‐function experiments showed that AMPK limits the induction of ferroptosis, and consequently, PF‐3758309 treatment restores the sensitivity toward ferroptosis inducers. The work established a chemical scaffold for the development of specific AMPK‐targeting compounds and deciphered the framework for the development of AMPK inhibitor‐based combination therapies tailored for PDAC.

## Graphical abstract

The metabolic orchestrator AMP‐activated protein kinase (AMPK) exhibits high expression in a subset of PDAC cases. Employing drug screening and drug target deconvolution a novel AMPK inhibitor is defined. This inhibitor exhibits efficacy in preclinical pancreatic cancer models and has the potential to sensitize cells to ferroptosis induction, thereby paving the way for novel translational concepts.

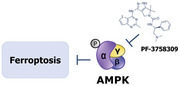

## 1 Introduction

The cyclic AMP‐dependent protein kinase (AMPK) is a crucial enzyme involved in the regulation of cellular energy homeostasis. It is a heterotrimeric serine‐threonine kinase composed of the catalytic subunit AMPKα and the regulatory subunits AMPKβ and AMPKγ, which are encoded by the genes *PRKAA1*, *PRKAA2*, *PRKAB1, PRKAB2*, *PRKAG1*, *PRKAG2, and PRKAG3*.^[^ ^1^, ^2^ ^]^ AMPK constantly monitors the ratios of adenosine monophosphate (AMP) to adenosine triphosphate (ATP) or adenosine diphosphate (ADP) to ATP, serving as a critical sensor of cellular energy status. While AMP:ATP ratio changes are the primary activators of AMPK through canonical pathways, non‐canonical pathways involving glucose starvation,^[^ ^3^ ^]^ DNA damage,^[^ ^4^, ^5^ ^]^ Ca2+/calmodulin‐dependent kinase CaMKK2,^[^ ^6^, ^7^, ^8^ ^]^ or transforming growth factor‐β‐activated kinase 1 (TAK1)^[^ ^9^ ^]^ have also been described, underscoring its role as broad homeostatic regulator.

The kinase plays a crucial role in balancing various cellular processes, including the inactivation of energy‐consuming pathways and the adaptive control of metabolic programs, like lipogenesis, glycolysis, or the citric acid cycle.^[^ ^1^ ^]^ AMPK is activated via phosphorylation of Thr172 by upstream kinases and allosteric activation induced by AMP binding to the γ subunit. The discovery that AMPK can be phosphorylated by the upstream kinase and tumor suppressor LKB1 (encoded by *STK11*) initially led to the belief that AMPK possesses tumor‐suppressive properties.^[^ ^1^, ^2^ ^]^ However, compelling genetic evidence in T‐cell acute lymphoblastic leukemia (T‐ALL) demonstrated that in established tumors, targeting *Prkaa1* can be a therapeutic approach.^[^ ^10^ ^]^ Moreover, it has been observed that cancer cells engage the AMPK pathway to cope with various cell‐intrinsic as well as extrinsic stresses.^[^ ^2^ ^]^ Therefore, AMPK inhibitors (AMPKi) hold promise as a concept to disrupt stress‐induced metabolic adaptability, offering prospects for novel therapies.^[^ ^11^ ^]^

The first and most widely used AMPKi is the ATP competitive inhibitor compound C or dorsomorphin.^[^ ^12^ ^]^ However, the inhibitor is rather a broad‐spectrum kinase inhibitor, targeting additionally CAMKK beta, CK1 delta, CLK2, DYRK1A, ERK8, GCK, IR, MELK, NUAK1, PHK, RIPK2, TrkA, VEGFR1, and YES1 with equal or greater potency (https://www.kinase‐screen.mrc.ac.uk/kinase‐inhibitors). SU6656, developed as an SRC kinase inhibitor, binds and inhibits the catalytic site of AMPK but also promotes paradoxical phosphorylation of Thr172.^[^ ^13^ ^]^ The dihydroxyquinoline MT47‐100 can activate or inhibit AMPK complexes.^[^ ^14^ ^]^ SBI‐0206965, developed as an Unc‐51‐like autophagy activating kinase 1 (ULK1) inhibitor,^[^ ^15^ ^]^ blocks AMPK downstream signaling.^[^ ^16^ ^]^ However, in cellular assay SBI‐0206965 concentrations >5 µM are needed to block AMPK signaling.^[^ ^16^ ^]^ Furthermore, the scaffold of the multi‐kinase inhibitor sunitinib was used to develop AMPK inhibitors, but these showed no impact on the cellular viability of leukemic K562 cells.^[^ ^17^ ^]^ Recently, BAY‐3827 was reported as a potent AMPKi. In cell‐based assays, BAY‐3827 was especially effective in prostatic cancer models.^[^ ^18^ ^]^ However, all of the above‐mentioned compounds are considered experimental drugs at this stage and have not been tested in clinical trials.

Taking into account the substantial costs, high failure rates, and lengthy timelines associated with developing new drugs, our objective was to find AMPKi inhibitors in an advanced developmental stage with the potential for accelerated development.^[^ ^19^ ^]^ This endeavor holds particular significance due to the urgent medical need for novel therapies in addressing pancreatic ductal adenocarcinoma. (PDAC).^[^ ^20^, ^21^, ^22^ ^]^ The critical involvement of metabolic rewiring in driving the progression of PDAC tumors^[^ ^23^ ^]^ accentuates the potential of targeting central metabolic networks and their orchestrators, including AMPK, in this disease. Thus, we elucidate the potential of repurposing the p21‐activated kinase 4 (PAK4) inhibitor PF‐3758309 as an AMPK inhibitor, demonstrate its efficacy in pre‐clinical PDAC models, and identify synergistic combination therapies as a basis for a clinically translatable treatment concept.

## 2 Results and Discussion

### 2.1 Active Prkaa1 is Expressed in a Subset of PDACs

**Figure 1:**
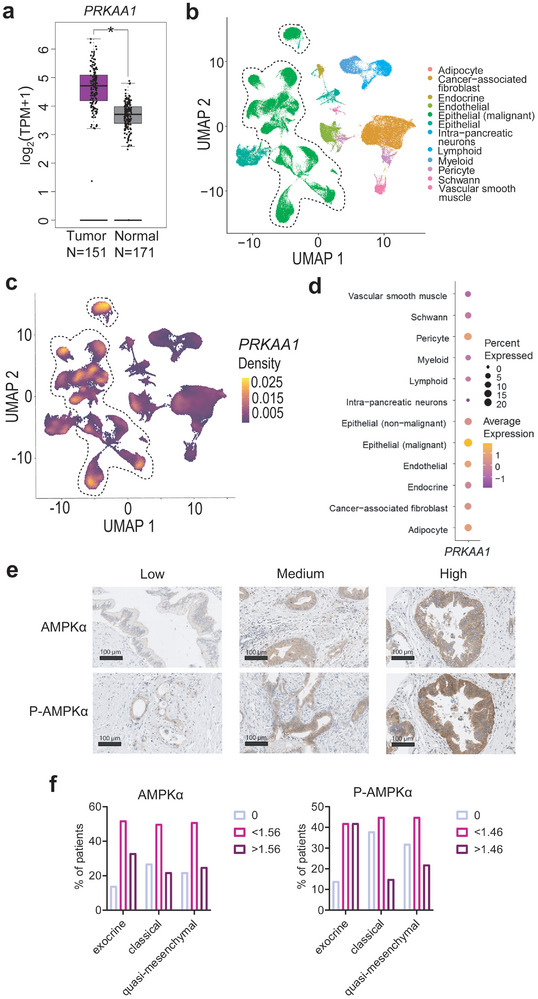
Prkaa1 is upregulated in a PDAC subtype. a) The PAAD dataset with curated 151 samples was matched to 171 GTEx samples by a GEPIA analysis (http://gepia2.cancer‐pku.cn/). Tissues are color‐coded (purple: Tumor, grey: Normal). mRNA expression is shown in log_2_(TPM+1). Log_2_FC cutoff: 0.58, *p*‐value cutoff: 0.05. PAAD: Pancreatic adenocarcinoma, GTEx: Genotype‐Tissue Expression project. b) UMAP of scRNA‐Seq of 18 PDAC patients with color‐coded cell types. Epithelial (malignant) cells are highlighted by a dashed line. N (cells) = 108917. c) Density plot of *PRKAA1* projected on UMAP of b. Density is color‐coded. d) Average expression of *PRKAA1* in cell types. Cell types are shown in rows and *PRKAA1* is shown in the column. The percentage (%) of cells expressing the gene is indicated by circle size. The maximum expression of the gene by cell type is color‐coded. e) Immunohistochemistry staining of P‐AMPKα and AMPKα in a cohort of 107 PDAC patients. Intensities of stained PDAC cells include low, medium, and high. f) Quantification of P‐AMPKα and AMPKα stained PDACs in exocrine (N = 21), classical (N = 44), and quasi‐mesenchymal (N = 31) subtypes. The percentage (%) of PDAC patients with specific subtypes and staining intensity is shown. Staining intensities are divided into three levels using a cut‐off finder (AMPKα: 0, <1.56, >1.56; P‐AMPKα: 0, <1.46, >1.46). Statistical analysis was performed by chi‐squared test.

To investigate the expression of *PRKAA1* in PDAC, we analyzed multiple datasets. Compared to the normal pancreas, upregulation of *PRKAA1* in cancer was observed in the GEPIA analysis of the PAAD dataset (**Figure** **1**a). To further validate our findings, we analyzed single‐cell RNA sequencing (scRNA‐Seq) data.^[^ ^24^ ^]^ The dataset comprised 108917 cells obtained from surgical samples of 18 untreated PDAC patients (Figure 1b). By examining *PRKAA1* mRNA expression across various cell types, we observed that malignant epithelial cells exhibited the highest average expression levels (Figure 1b–d), pointing to a major tumor cell‐intrinsic function of the kinase. To corroborate the increased expression of AMPKα at the protein level, we used immunohistochemistry. We stained a cohort of 107 resected PDACs using phospho (T172) (p)‐AMPKα‐ and AMPKα‐specific antibodies. Consistent with the scRNA‐seq data, a cancer‐cell‐specific staining pattern was observed (Figure 1e). Interestingly, the pattern was highly variable, from negative staining to PDACs with a high abundance of phospho‐ and AMPKα staining (Figure 1e,f; Figure S1a, Supporting Information). However, we did not observe a statistically significant association between staining intensity and a specific PDAC subtype (Figure 1f), grade, or survival (Figure S1b,c, Supporting Information). In sum, we observed high expression and phosphorylation of AMPKα in a subset of PDACs.

### 2.2 Prkaa1 is Associated with a Metastatic and Mesenchymal PDAC Phenotype

**Figure 2:**
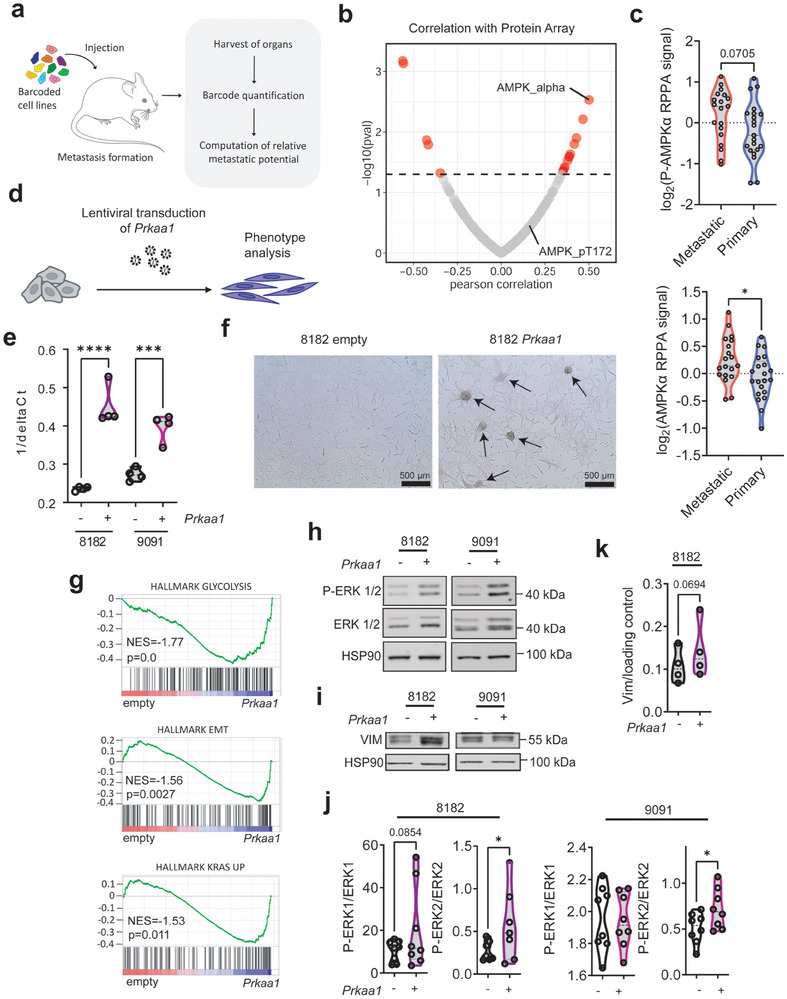
*Prkaa1* expression is correlated with de‐differentiation and metastasis. Data of a, b, and c, were accessed via https://depmap.org/. a) Scheme of in vivo barcoding strategy to determine the metastatic potential of human cancer cell lines in mouse xenografts. Cancer cell lines were barcoded, pooled, and injected into immunodeficient mice. After metastatic growth, organs were harvested, and DNA barcodes were quantified by next‐generation sequencing. The metastatic potential of each cell was quantified as barcode enrichment relative to the abundance in the pre‐injected population. b) Correlation of protein array data (N(Antibodies) = 214) with metastatic potential in human PDAC cell lines. On the x‐axis, the Pearson correlation coefficient is shown. On the y‐axis ‐log(pval) is shown. Cut‐off:1.3. c) log_2_(RPPA signal) of P‐AMPKα at Thr172 and AMPKα in primary and established pancreatic cancer cell lines derived from primary tumors or metastatic sites. Statistical analysis was performed by a two‐tailed unpaired *t*‐test. d) Scheme of generation of *Prkaa1* overexpressing cell lines. Cells were generated by lentiviral transduction of a PGK‐Vector expressing *Prkaa1*. e) qPCR of *Prkaa1* mRNA in empty vector control (−) and *Prkaa1* vector (+) transduced cells. The quantity of *Prkaa1* expression is shown on the x‐axis as 1/delta Ct. Statistical analysis was performed by one‐way ANOVA with Bonferroni correction. f) Microscopic pictures of 8182 PDAC cells transduced with empty vector control or the *Prkaa1* vector. The scale bar is shown in the bottom left. Arrowhead: spheroid growth pattern. g) GSEA of RNA‐Seq data in 8182 empty versus 8182 *Prkaa1* cells using the HALLMARK gene set database. Normalized enrichment scores (NES) and *p*‐values (p) are shown. Western Blots of ERK pathway h) and Vimentin i) in empty (−) and *Prkaa1* (+) overexpressing cells. ERK pathway was investigated using P‐ERK1/2 and ERK1/2 antibodies. HSP90 was used as loading control. The same lysates were transferred to two membranes and subsequently, incubated with either pan or phospho‐antibodies and used to determine the relative phosphorylation level of protein of interest. j) Quantification of h. Statistical analysis was performed by a one‐tailed unpaired *t*‐test. k) Quantification of i. Statistical analysis was performed by a one‐tailed unpaired *t*‐test. EMT: Epithelial‐to‐mesenchymal transition, ERK: extracellular signal‐regulated kinase, GSEA: Gene set enrichment analysis, P‐: Phosphorylation, RPPA: Reverse phase protein array, *p < 0.05, **p < 0.01, ****p < 0.0001.

To further explore the role of AMPK in PDAC, we conducted an analysis utilizing data from the DepMap portal. This platform provides access to information regarding the metastatic capabilities of human cancer cell lines.^[^ ^25^ ^]^ A barcoding strategy was used by these authors to evaluate the metastatic growth of cell lines, which was the basis for the computation of a metastatic potential score (**Figure** **2**a). Through the correlation of the metastatic potential of human PDAC cell lines with protein array data, our analysis revealed that AMPKα exhibited the highest Pearson correlation coefficient with the metastatic potential of PDAC cells (R = 0.51) out of the 214 antibodies of selected proteins and phosphorylation sites included (Figure 2b). No correlation between the phosphorylation of AMPKα and the metastatic potential was detected (Figure 2b). To further substantiate the link of AMPK to metastasis, we accessed proteomic data^[^ ^26^ ^]^ and observed a correlation between the relative metastatic potential of human PDAC lines and all AMPK subunits, except for *PRKAA2*, which exhibited low expression (Figure S2a, Supporting Information). Furthermore, in the comparison of PDAC cell lines derived from primary tumors and those originating from a metastatic site, metastatic PDAC cell lines exhibited elevated AMPKα expression and phosphorylation (Figure 2c).

In addition to the established human PDAC cells, we expanded our dataset by incorporating primary murine PDAC cells, derived from KRAS^G12D^‐driven genetically engineered PDACs.^[^ ^27^ ^]^ We used transcriptome profiles of 38 murine PDAC cell lines and clustered them based on their AMPK subunit mRNA expression (Figure S2b, Supporting Information). We consistently observed an association between elevated *Prkaa1* expression and cell lines established from undifferentiated cancers, an association with metastasis, and a mesenchymal phenotype of the lines (Figure S2c–f, Supporting Information).

To experimentally connect *PRKAA1* to dedifferentiation, we generated overexpressing murine PDAC cell lines using lentiviral transduction (Figure 2d). We chose two murine PDAC cell lines, 8182 cells, a more epithelial cell line from the AMPK‐Low cluster, and 9091, an undifferentiated mesenchymal cell line from the AMPK‐High‐1 cluster (Figure S2b, Supporting Information). We validated *Prkaa1* overexpression by qPCR (Figure 2e) and by western blot using p‐AMPKα, and AMPKα antibodies (Figure S3a–c, Supporting Information). Furthermore, AMPK downstream signaling using p‐ACC and ACC western blots was investigated (Figure S3a–c, Supporting Information). Increased *Prkaa1* expression does not impact the short‐term proliferative capacity of PDAC cells (Figure S3d, Supporting Information). Interestingly, we observed that *Prkaa1* overexpressing 8182 cells at low passages exhibited clusters with spherical growth patterns (Figure 2f; Figure S4, Supporting Information). To further explore the function of *Prkaa1*, we performed RNA‐Seq analysis of overexpressing cell lines and their controls. The transcriptional profiles were analyzed using gene set enrichment analysis (GSEA). Notably, 8182 *Prkaa1* overexpressing cells exhibited enrichment of the MSigDB Hallmark pathways glycolysis, epithelial‐to‐mesenchymal transition (EMT), and KRAS UP (Figure 2g). EMT is a process in which differentiated epithelial cells acquire mesenchymal characteristics. In cancer, EMT is associated with a stem‐cell‐like phenotype and metastasis.^[^ ^28^ ^]^ Indeed, AMPK overexpression was connected to increased expression of the mesenchymal marker Vimentin (Figure 2i,k) and activation of the canonical KRAS pathway, as detected by increased phosphorylation of ERK1/2 (Figure 2h,j; Figure S5a, Supporting Information), substantiating our transcriptomic data. To confirm our findings, we accessed several datasets. In protein array data from the DepMap portal, we found a weak but significant correlation between MAPK1 (ERK2)/MAPK3 (ERK1) phosphorylation and AMPKα expression across all cancer types, although not significant in PDAC (Figure S5b, Supporting Information). However, RNA‐Seq data from The Cancer Genome Atlas (TCGA), the International Cancer Genome Consortium (ICGC), the DepMap portal, and published in^[^ ^29^ ^]^ showed a correlation between *PRKAA1* and *KRAS, MAPK3*, and *MAPK1* expression (Figure S5c, Supporting Information). Overall, these data indicate that PRKAA1 correlates with a less differentiated PDAC phenotype and activity of the RAS‐MEK‐ERK signaling pathway.

### 2.3 A Drug Screen Uncovers a Potential AMPKi

So far, our results identified an association of AMPK with dedifferentiated and metastatic phenotypes. Therefore, we propose that the targeting of AMPK could potentially serve as a therapeutic strategy. To identify novel AMPKi, we employed a drug repurposing screening approach.

**Figure 3:**
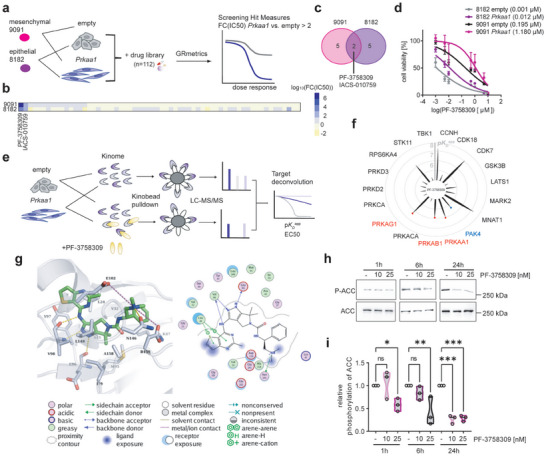
The drug screen identifies PF‐3758309 as AMPKi. a) Experimental setup of drug screen in empty vector control and *Prkaa1* overexpressing PDAC cell lines. 9091 and 8182 empty and *Prkaa1* cell lines were treated with a 7‐fold dilution of a drug library containing 112 compounds under clinical testing. After 72 h, cell viability was measured and dose‐response curves were generated by applying the GRmetrics package. Screening hits were defined as fold change (inhibitory concentration 50) (FC(IC_50_)) of *Prkaa1* versus empty >2. b) Heatmap of log_10_(FC(IC_50_)) in 9091 and 8182 cells. c) Venn diagram of screening hits in 9091 and 8182 cells. d) Cell viability measurement of empty and *Prkaa1* cells treated with PF‐3758309 in a seven‐fold dilution series for 72 h. The Y‐axis shows cell viability in percent [%] normalized to DMSO control. X‐axis shows concentration of PF‐3758309 as log_2_(PF‐3758309 [µm]). IC_50_ values are shown in brackets. Experiments were performed as three technical replicates with four biological replicates. e) Target deconvolution strategy using the kinobeads assay. The kinome of empty and *Prkaa1* overexpressing cells was pulled down using kinobeads either in the presence of different concentrations of PF‐3758309 or without. Kinases were detected via LC‐MS/MS. Targets were ranked according to the apparent dissociation constant *K_D_^app^* and effective concentration 50 (EC_50_). f, Radar plot of identified targets of PF‐3758309 and their negative log_10_(*K_D_^app^*) (p*K_D_^app^*). Spike length indicates p*K_D_^app^* of the respective kinase. AMPK subunits are indicated in red. The intended target Pak4 is indicated in blue. g) Interaction of AMPKα (PDB ID 6C9F) with cocrystallized PF‐03758309. Right: 2D plot of kinase‐ligand interaction. Left: 3D representation of the binding of the inhibitor (colored green) to the ATP pocket. Hydrogen bonds are shown as yellow‐colored dashed lines. Salt bridges are shown as magenta‐colored dashed lines. Water molecules are displayed as red spheres. h) Western blot and i) quantification of AMPK signaling upon PF‐3758309 treatment. Cells were treated with 10 or 20 nM of PF‐3758309 or left as vehicle‐treated controls. Protein was harvested after 1, 6, and 24 h. AMPK inhibition was investigated by P‐ACC and ACC antibodies. P‐ACC was normalized to ACC. Hsp90 served as loading control. Experiments were performed as three biological replicates. ACC: Acetyl‐CoA carboxylase, Hsp90: Heat shock protein 90, P‐: Phosphorylation, ns: not significant, *p < 0.05, **p < 0.01, ***p < 0.005.

We performed a systematic compound screen using two isogenic *Prkaa1* gain‐of‐function models (**Figure** **3**a). These cell lines were subjected to treatment with a panel of drugs (n = 112, Table S3, Supporting Information) currently undergoing preclinical and clinical investigation. As a result of the screening, we identified seven hits in each pair of cell lines (Tables S4,S5, Supporting Information). A Venn analysis revealed two common hits, namely PF‐3758309 and IACS‐010759 (Figure 3b,c), both exhibiting diminished activity in cells overexpressing *Prkaa1*. IACS‐010759 is a quinone‐site inhibitor of the oxidative phosphorylation complex I.^[^ ^30^ ^]^ However, since our objective was to identify a potential AMPKi, our focus was on the kinase inhibitor PF‐3758309. Originally developed as an inhibitor of p21‐activated kinase 4 (PAK4), PF‐3758309 has demonstrated significant anti‐tumor activity in in vivo models.^[^ ^31^ ^]^ Furthermore, PF‐3758309 has already undergone a phase I clinical trial (NCT00932126). Reduced PF‐3758309 activity in *Prkaa1* gain‐of‐function models could be validated in viability assays (Figure 3d) and long‐term clonogenic growth assays (Figure S6a,b, Supporting Information). Furthermore, the resistance of cells with high *Prkaa1* expression toward PF‐3758309 was confirmed in the Cancer Target Discovery and Development (CTD2) screening data^[^ ^32^ ^]^ accessed via the DepMap portal (Figure S6c, Supporting Information).

To validate the potential AMPK inhibitory activity of PF‐3758309, we employed the kinobeads assay,^[^ ^33^ ^]^ enabling a comprehensive assessment of inhibitor‐protein interactions (Figure 3e). In this analysis, we utilized 8182 cells, including both, empty vector controls and *Prkaa1* overexpressing cells. We identified overlapping target kinases based on specific filtering criteria: effective concentration 50 (EC_50_) < 500 nM, R^2^ > 0.8, and bottom of curve <0.2. All AMPK subunits were detected, alongside the intended target PAK4 for PF‐3758309 (Figure 3f). Remarkably, the affinity of PF‐3758309 toward the AMPK subunits was found to be higher than that toward PAK4. Moreover, PF‐3758309 exhibited the highest affinity for PRKAA1 among the 243 kinase inhibitors profiled in https://www.proteomicsdb.org/ ^[^ ^34^ ^]^ (Figure S6d, Supporting Information). Our data align with the previous report by Murray et al. estimating an inhibitory constant (K) of 5 nm for AMPK in kinase activity in vitro assay of PF‐3758309.^[^ ^31^ ^]^ Furthermore, when correlating the PF‐3758309 response with potential other target, like CDK7, only PRKAA1 demonstrated a significant correlation (Figures S6c, S7, Supporting Information). Consistently, PAK4‐deficient cells displayed analogous responses to PF‐3758309 compared to their proficient counterparts.^[^ ^35^ ^]^ Taken together, this data supports a PAK4‐independent mode of action of PF‐3758309 and points to an alternate target.

To analyze the binding of PF‐3758309 to key kinases identified in the kinobeads assay, in silico docking experiments were performed with the kinases showing the highest pEC_50_ namely, AMPKα, CDK7, and PAK4 (Figure 3g; Figure S8, Supporting Information). Among the 48 available AMPKα crystal and cryoEM structures, only three (PDB structures 4RER, 6C9F, and 6C9H) exhibited a docking solution analogous to the binding mode of PF‐3758309 in PAK4 (PDB ID 2×4Z). In AMPKα, PF‐3758309 binds to the ATP pocket, forming hydrogen bonds with Glu96 and Val98, similar to PAK4 (Figure 3g). Additionally, hydrophobic interactions were observed with other residues. Similar binding modes were also observed in the docking pose of PF‐3758309 with CDK7 (Figure S8, Supporting Information). The observed energetically favorable interactions in the studied kinase structures are in good agreement with the experimentally determined binding and might be used to further optimize the inhibitor for AMPK.

To provide evidence that PF‐3758309 not only binds to AMPK but also inhibits its downstream signaling, we investigated the phosphorylation of ACC, a well‐established target of AMPK, using western blot analysis (Figure 3h,i). Our results demonstrate a notable inhibition of AMPK signaling, as indicated by the suppression of ACC phosphorylation. Importantly, this inhibition occurred within a concentration range of 10–20 nm similar to that observed in the kinobeads assay, further supporting the inhibitory effects of PF‐3758309 on AMPK.

### 2.4 PF‐3758309 is Effective in Preclinical PDAC Models

**Figure 4:**
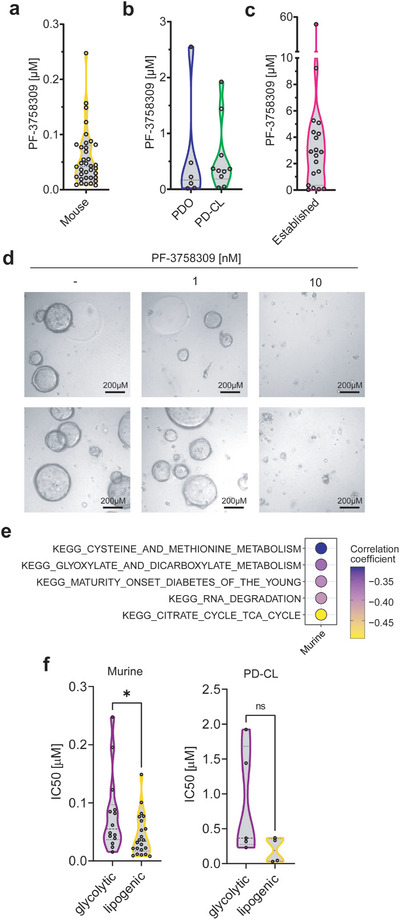
PF‐3758309 is effective across PDAC models. a) Half‐maximal Inhibitory concentration (IC_50_) of PF‐3758309 after 3 days of treatment in murine cell lines (N = 37) using CellTiter‐Glo. b) IC_50_ of PF‐3758309 in patient‐derived organoids (PDO) (N = 6), patient‐derived cell lines (PD‐CL) (N = 9) using CellTiter‐Glo. c) IC_50_ of PF‐3758309 in established human PDAC (N = 19) cell lines derived from CTD2 screen accessed via Depmap. d) Microscopic pictures of PDOs treated with different concentrations of PF‐3758309 after 7 days. The scale bar is shown in the bottom right. Two biological replicates are depicted. e) Pearson correlation coefficient of GSVA of KEGG gene sets and IC_50_ of PF‐3758309 in murine cell lines. f) IC_50_ of PF‐3758309 of murine and patient‐derived cell lines (PD‐CL) (N = 9) in glycolytic and lipogenic PDAC subtypes. Differential genes for clustering were selected based on adjusted *p*‐value < 0.05 and log_2_(fold change) >1.

To evaluate the potential efficacy of PF‐3758309 against PDAC, we determined IC_50_ values of various PDAC models using a 7‐point drug dilutions after three days of treatment (**Figure** **4**a,b). Our study encompassed a comprehensive panel of 37 murine cell lines, 9 patient‐derived cell lines (PD‐CLs), and 6 organoids (PDOs) (Table S9, Supporting Information). Furthermore, we accessed the CTD^2^ screen via Depmap to investigate the dose‐response of established PDAC cell lines (n = 19) (Figure 4c; Table S9, Supporting Information). Especially, the primary human models as well as the murine cell lines demonstrated significant sensitivity to PF‐3758309, with IC_50_ in the double‐digit to single‐digit nM range in some lines. Notably, in organoids, prolonged exposure to PF‐3758309 resulted in enhanced efficacy, as complete loss of viability was observed at a concentration as low as 10 nm of the compound (Figure 4b,d). When comparing the sensitivity of cell lines derived from metastatic or primary sites, we did observe similar responses (Figure S9a,b, Supporting Information). To determine potential biomarkers for PF‐3758309 responsiveness, we conducted single sample gene set variation analysis (ssGSVA) of the KEGG gene sets in the panel of the primary murine cell lines, and found KEGG_CITRATE_CYCLE_TCA_CYCLE to be the top gene set that negatively correlated with sensitivity to PF‐3758309 (Figure 4e). Moreover, we integrated the gene signature from Daemen et al., which used metabolite profiling to determine a so‐called lipogenic and glycolytic subtype.^[^ ^36^ ^]^ Leveraging their metabolite data, they identified differentially expressed genes within these subtypes, which we subsequently employed for classification. Remarkably, we found that cell lines categorized as lipogenic exhibited a heightened sensitivity to PF‐3758309 (Figure 4f), pointing to the possibility of patient stratification. These findings suggest the presence of a potential metabolic vulnerability in a subset of PDAC cells that could be targeted by PF‐3758309. Overall, PF‐3758309 shows efficacy across preclinical PDAC models.

### 2.5 PF‐3758309 Controls Metabolic Pathways

To comprehensively investigate the pathway downstream of PF‐3758309, we treated 8182 and 9091 empty cells with PF‐3758309 for 24 h and performed RNA‐Seq (Figure S10a, Supporting Information). We then used Genetrail 3.2 to analyze the enrichment of KEGG gene sets (Release 109.0).^[^ ^37^ ^]^ Our research revealed a concurrent enrichment of autophagic and lysosomal signatures upon treatment, while ribosomal signatures emerged as the most prominently downregulated in both cell lines (Figure S10b–e, Supporting Information). Additionally, we observed further downregulation in signatures associated with splicing, cell cycle, and DNA replication. To validate the RNA‐Seq results, we performed Western Blotting of the autophagy marker LC3BI/II and found a slight elevation following 24 h of PF‐3758309 treatment (Figure S11a,b, Supporting Information). Given AMPK's known role in regulating glucose uptake, we employed a Seahorse assay after subjecting cells to a 6‐h treatment with PF‐3758309 (Figure S11c, Supporting Information). Our observations unveiled a decrease in extracellular acidification, suggesting a reduction in glycolytic activity upon PF‐3758309 treatment.

### 2.6 AMPK is Connected to MEK Inhibitor Sensitivity

Since we detected the connection of AMPK to canonical KRAS signaling, we investigated the efficacy of the MEK inhibitor (MEKi) Trametinib. We conducted a combination treatment over a 7‐day period using both 8182 control cells and cells overexpressing *Prkaa1* (Figure S12a, Supporting Information). Intriguingly, our findings revealed that cells overexpressing *Prkaa1* exhibited lower sensitivity to Trametinib (Figure S12b, Supporting Information). Furthermore, we observed the synergy of PF‐3758309 with Trametinib (Figure S12a,c, Supporting Information). These observations are consistent with our recent findings that AMPK contributes to MEKi resistance in PDAC.^[^ ^38^ ^]^

### 2.7 Prkaa1 Knock‐Out Triggers Collateral Vulnerabilities

To recapitulate the PF‐3758309 effect concerning AMPK inhibition on PDAC, we used CRISPR Cas9 *Prkaa1* knock‐out (KO) cell lines. We used three *Prkaa1* KO cell lines from the AMPK‐High‐1 cluster (8570, 9091, and 8248), which we have recently generated^[^ ^38^ ^]^ (Figure S13a, Supporting Information). Two different sgRNAs targeting *Prkaa1* (KO1, KO2) and LacZ sgRNA as a control were used. The successful knock‐out of *Prkaa1* was confirmed through western blot analysis of AMPKα and phosphorylation of its downstream target ACC (Figure S13b,c, Supporting Information). Surprisingly, contrary to our initial expectations, the growth rates of *Prkaa1* KO cells were found to be similar to those of the LacZ control cells (Figure S13d, Supporting Information). This suggests long‐term loss of *Prkaa1* can be compensated with respect to the overall proliferative capacity. However, in‐depth investigation of cellular fitness in *Prkaa1* KO cells by assessment of basal caspase activity, we observed an induction of Caspase 3/7 activity in 8248 and 9091 *Prkaa1* KO cells (Figure S14a, Supporting Information), suggesting a concealed dysregulation in these cell lines.

To further characterize the *Prkaa1* knock‐out, we performed an RNA‐Seq experiment as well as Seahorse assays. We found significant changes in gene expression in the 8248 and 9091 *Prkaa1* KO cells, but not in the 8570 *Prkaa1* KO cells (Figure S14b, Supporting Information). Consistent with our previous finding that AMPK was connected to dedifferentiation, the EMT signature was depleted in *Prkaa1*‐deficient cells (Figure S14c, Supporting Information). Surprisingly, the *Prkaa1* KO cells exhibited only minor changes in glycolysis and oxidative phosphorylation parameters determined by the Seahorse assay, and no consistent pattern was determined across all cell lines (Figure S14d, Supporting Information). To further substantiate the connection between AMPK and cellular capabilities which might be connected to EMT and metastasis, we conducted an organoid branching assay utilizing 9091 LacZ control and *Prkaa1* knock‐out cells, namely KO1and KO2 (Figure S15, Supporting Information). Here, we observed a decrease in branching capabilities for both KO1 and KO2 (Figure S15a, Supporting Information). Extending the observation period to 13 days and conducting a floating collagen gel assay,^[^ ^39^ ^]^ we observed firework‐like and dense branched phenotypes in 9091 LacZ control cells, whereas KO1 and KO2 cells formed slender branched and loosely clustered colonies (Figure S16, Supporting Information). In sum, our data connect AMPK to cellular branching phenotypes.

In addition, we used the AMPK knock‐out cell lines to evaluate the contribution of the kinase to the PF‐3758309 response. Especially at low doses, the PF‐3758309 response is alleviated (Figure S17, Supporting Information).

In sum, we concluded that I) *Prkaa1* knock‐out can be compensated however, II) the increased Caspase 3/7 activity points to a potential collateral vulnerability of *Prkaa1*‐deficient cells, and III) AMPK inhibition is relevant for the PF‐3758309 response.

### 2.8 Potential AMPKi Based Combination Therapies

**Figure 5:**
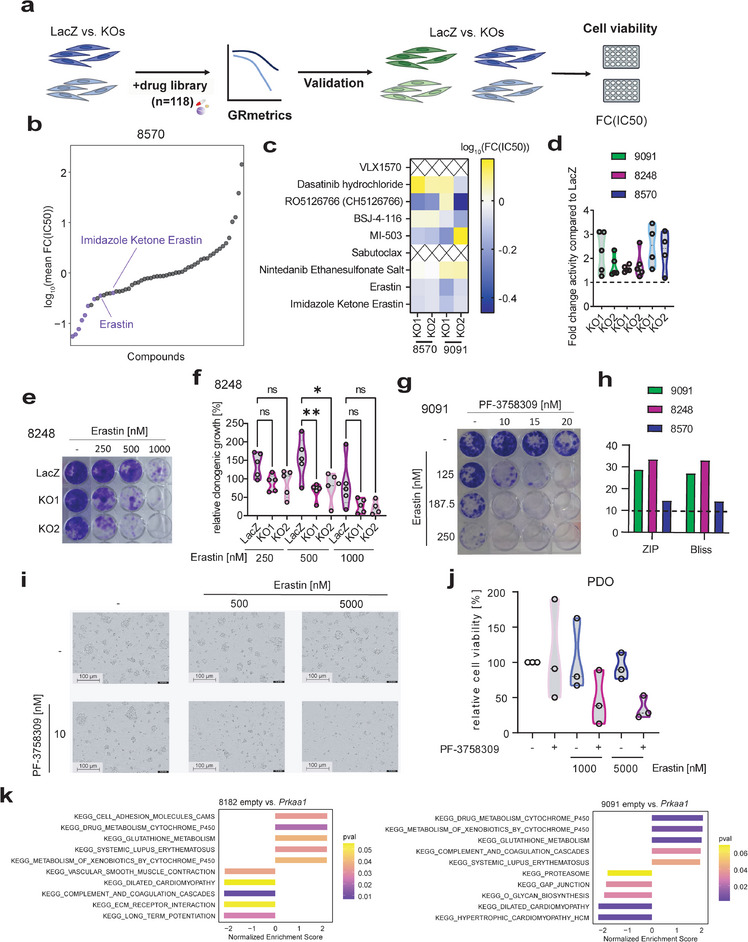
Drug Screen to uncover *Prkaa1*‐dependent processes and associated vulnerabilities. a) Scheme of a drug screen and validation workflow. 8570 LacZ control cells (dark blue) and corresponding *Prkaa1* KO1, and *Prkaa1* KO2 cells (light blue) were screened with a drug library containing 118 compounds. Hits were defined based on FC(IC_50_) < 0.7 in both KOs. Potential hits were first validated in LacZ control cells and *Prkaa1* KOs of 8570 and subsequently in LacZ control cells (dark green) and Prkaa1 KOs (light green) of 9091 cells. b) Dot plot of log_10_ (mean fold change inhibitory concentration 50 (FC(IC_50_))) in 8570 LacZ control cells versus *Prkaa1* KOs. Drugs are ordered according to their mean FC(IC_50_). Highlighted are drugs with FC(IC_50_) < 0.7 in both KOs and the top hit Erastin as well as its analog Imidazole Ketone Erastin. c) Screening hits of b were validated in LacZ control versus *Prkaa1* KO1 and *Prkaa1* KO2 of 8570 and 9091 cells after 72 h of treatment by CellTiter‐Glo^®^ assay (N = 3). Cell viability was normalized to DMSO control. X‐axis shows concentration of PF‐3758309 as log_2_(PF‐3758309[µM]). Experiments were performed as three technical replicates with three biological replicates. Log_10_(FC(IC_50_))) was calculated and color‐coded. X indicates false positive hits. d) Fold change activity of Erastin in *Prkaa1* KOs compared to LacZ control cells. Relative clonogenic growth of LacZ control cells treated with Erastin was divided by relative clonogenic growth of *Prkaa1* KO cells treated with Erastin. Resulting value is depicted as fold change activity. Experiments were performed at least as four biological replicates. e) Clonogenic assay of 8248 LacZ control cells and corresponding *Prkaa1* KO1, and *Prkaa1* KO2 treated with indicated concentrations of Erastin for 8 days. Experiments were performed as five biological replicates. f) Quantification of e. g) Clonogenic assay of 9091 LacZ control cells treated with indicated concentrations of Erastin and PF‐3758309 for 8 days. h) Synergy scores of combination treatment of Erastin and PF‐3758309 in 8570, 8248, and 9091 LacZ control cells. Zero interaction potency (ZIP) and Bliss scores for each cell line are shown. Experiments were performed as at least four biological replicates. i) Microscopic images of PDOs treated with indicated concentrations of PF‐3758309 and Erastin for 7 days. j) Cell viability of PDOs treated with indicated concentrations of PF‐3758309 and Erastin for 6 days. k) GSEA of RNA‐Seq data of empty versus *Prkaa1* overexpressing cells using the KEGG gene set database. Depicted are the top 5 enriched and depleted KEGG gene sets. Normalized enrichment scores are shown on the x‐axis and *p*‐values (pval) are color‐coded.

Building upon our identification of a potential hidden vulnerability in the absence of AMPK, our objective was to translate this discovery into a prospective rational PDAC‐tailored combination therapy. Moreover, the implementation of a combination therapy would empower us to overcome the adaptive changes associated with *Prkaa1* loss and effectively counteract the described resistance driven by AMPK against PF‐3758309. We used a two‐step approach to determine a true collateral AMPK knock‐out‐associated vulnerability. First, we utilized *Prkaa1* KO cell lines and subsequently validated the identified combinations using PF‐3758309. Therefore, we again designed a drug screening experiment. We used 8570 LacZ control cells, KO1, and KO2 cells and treated them with n = 118 drugs (**Figure** **5**a). We determined screening hits in this PDAC line (Figure 5b; Table S6, Supporting Information) and additionally validated them in the 9091 LacZ control cells and corresponding *Prkaa1* KO1 and KO2 models. In such a cross‐over design, only one therapeutic principle was connected to AMPK – Ferroptosis induction by Erastin (Figure 5c). These findings highlight the high level of heterogeneity observed in PDAC, while also emphasizing the crucial role of *Prkaa1* in preventing Erastin‐induced ferroptosis within the context of PDAC. We validated these findings in all three KO cell lines in long‐term clonogenic growth assays (Figure 5d–f; Figure S18a–d, Supporting Information) and performed combination therapies with PF‐3758309 which revealed a synergistic interaction (Figure 5g,h; Figure S18e,f, Supporting Information). In order to validate that the combination therapy is also effective in translational human models, we treated two patient‐derived organoid (PDO) lines with the combination therapy and found effective responses in both (Figure 5i,j; Figure S18g,h, Supporting Information). To investigate the effect on non‐cancerous cells, we utilized immortalized human HaCaT keratinocytes and exposed them to treatment with Erastin and PF‐3758309 for a duration of 7 days (Figure S19a,b, Supporting Information). Our investigation revealed that while PF‐3758309 monotherapy did impact clonogenic growth, the combined therapy did not yield any supplementary effects. These findings suggest the potential presence of a therapeutic window for combination therapy. To characterize the underpinnings of the connection of AMPK to ferroptosis, we analyzed our RNA‐Seq data. In *Prkaa1*‐gain‐of‐function models, we detected enrichment of signatures associated with ROS detoxification and defense, like glutathione metabolism, or drug metabolism (Figure 5k). In the genetic loss‐of‐function models, these signatures were modulated but inconsistently linked to AMPK (Figure S20, Supporting Information). However, given that the signature of similar pathways was among the top 5 regulated signatures in all cell lines, these signatures suggest that PRKAA1 plays a role in drug metabolism and glutathione homeostasis in PDAC cells.

To explore genes directly linked to ferroptosis, we performed transcriptome analyses on 8182 and 9091 control cells treated with PF‐3758309 for 24 h, as well as on 9091 and 8248 LacZ control cells and corresponding *Prkaa1* KO1 and *Prkaa1* KO2 cells. These analyses revealed dysregulation of key genes implicated in oxidative stress, iron metabolism, glutathione metabolism, and autophagy (Figure S21a,b, Supporting Information). Exploring the possibility that AMPK inhibition directly initiates ferroptosis, we conducted experiments examining the effects of Liproxstatin‐1, an iron‐chelating agent and established ferroptosis inhibitor, as well as *N*‐Acetylcysteine, a precursor of glutathione (Figure S21c,d, Supporting Information). Our findings reveal that neither of these compounds changed the PF‐3758309 response significantly. This suggests that while PF‐3758309 increased the susceptibility to ferroptosis inducers, it does not directly initiate ferroptosis. To further enhance the evidence, we subjected 9091 control and *Prkaa1* KO cells to Erastin treatment for 24 h. Subsequently, we examined ferroptosis‐related proteins by western blot (Figure S21e–g, Supporting Information). We observed that Erastin indeed prompted increased upregulation of the ferroptosis markers TXNIP and HMOX1 in knock‐out cells compared to controls. Furthermore, at 48‐h of the combination therapy, we specifically assessed both total and oxidized glutathione levels (Figure S21h,i, Supporting Information). Although our findings indicated a slight increase in oxidized glutathione levels in the combination therapy, it also resulted in a significant depletion of total glutathione within the cells.

Overall, we found that genetic or pharmacological inhibition of AMPK renders PDAC cells sensitive to ferroptosis induction by Erastin.

## 3 Conclusion

The incidence and mortality of PDAC are increasing, and the 5‐year survival rate of 13% is frustratingly low.^[^ ^40^, ^41^ ^]^ The current standard of care for most patients remains modestly active combination chemotherapies,^[^ ^42^ ^]^ underscoring the need to develop additional therapeutic options. Considering the clear metabolic dependencies and metabolic adaptability of PDAC,^[^ ^43^ ^]^ we focused on AMPK, a central metabolic regulator. We show that AMPK expression and phosphorylation are increased in ≈20% of PDACs, provide evidence of a tumor‐promoting function, and show that AMPK limits the induction of ferroptosis.

PDAC can be subtyped based on its metabolic pathways. A lipid metabolism‐activated subtype that overlaps with the classical subtype and a glycolysis‐activated subtype that overlaps with the more aggressive basal‐like subtype were consistently determined.^[^ ^22^, ^36^, ^44^, ^45^ ^]^ A recent study found that the mRNA expression of *PRKAA1* is increased in lipogenic PDACs.^[^ ^44^ ^]^ While we did not find an association between AMPK protein expression or phosphorylation and a specific PDAC subtype in a PDAC patient cohort, we observed a potential link between AMPK expression, the EMT program in vitro, a metastatic phenotype, and the established driver pathway, the RAS‐MEK‐ERK signaling axis. Illustrated in a recent study employing scRNA‐Seq to explore human breast cancer metastasis, diverse metabolic strategies may provide cells with distinct advantages in traversing different stages of the metastatic cascade.^[^ ^46^ ^]^ This underscores the critical role of metabolic adaptability in facilitating these processes. Consistently, overexpression of AMPK has been linked to a shorter metastasis‐free survival of breast cancer patients.^[^ ^47^ ^]^ The authors confirmed their findings by knock‐down of PRKAA1 in 4T1 breast cancer cells which reduced the lung metastasis capabilities of the cells in vivo. Notably, in PDAC, AMPK shows the ability to interact and phosphorylate an essential inducer of EMT, ZEB1, especially in the presence of metabolic stress.^[^ ^48^, ^49^ ^]^ This underscores the role of AMPK in shaping the cellular phenotype and influencing key factors in the metastatic cascade. Furthermore, in the context of lung cancer, AMPK activation has been shown to confer resistance to anoikis and promote metastasis.^[^ ^50^ ^]^ Consistently, in vivo models of prostate cancer have shown that circulating tumor cells activate stress‐protective AMPK signaling, fostering metastatic capabilities.^[^ ^51^ ^]^ Within an alternative mutational background, particularly the loss of tumor suppressor *PTEN*, researchers have elucidated the involvement of LKB1‐dependent activation of AMPK in driving enhanced collective migration.^[^ ^52^ ^]^ Using the TCGA PDAC mRNA expression dataset, high AMPK mRNA expression was linked to a worse prognosis, and a connection of AMPK to glycolysis was described.^[^ ^53^ ^]^ While these studies demonstrate the role of AMPK in tumor progression and corroborate our findings, the data on AMPK's role in PDAC are conflicting. In contrast, despite robust AMPK expression in PDAC, a series of 72 PDACs demonstrated loss of AMPK phosphorylation in 83% of cases.^[^ ^54^ ^]^ Furthermore, phosphorylation of AMPK was connected to a better prognosis in this study,and indirect pharmacological activation by respiratory complex I inhibition of AMPK was connected to reduced invasion and migration.^[^ ^54^ ^]^ Furthermore, interfering with AMPK expression in PDAC cell lines using RNA interference was shown to increase invasion and migration.^[^ ^55^ ^]^ Such discrepancies may be explained by short‐term knock‐downs and the usage of non‐selective inhibitors/activators. In summary, further investigation is needed to clarify the context‐specific functions of AMPK in PDAC.

In our repurposing approach, we unbiasedly found and characterized the PAK4 inhibitor PF‐3758309^[^ ^31^ ^]^ as an AMPKi. Chemoproteomic target affinity profiling showed binding of PF‐3758309 to AMPK with EC_50_ values in the nanomolar range. Furthermore, a robust in silico docking model was elaborated for AMPK, and in PDAC cells, AMPK downstream signaling was reduced. Although the PF‐3758309 showed relevant in vivo activity in solid tumors,^[^ ^31^ ^]^ including PDAC,^[^ ^56^ ^]^ the phase I study was closed due to poor pharmacokinetic properties observed (NCT00932126, https://classic.clinicaltrials.gov/ct2/show/NCT00932126). Gastrointestinal adverse effects were the most relevant PF‐3758309 toxicities and no clinically relevant response was described.^[^ ^57^ ^]^ Therefore, we see PF‐3758309 as a scaffold to develop more specific AMPKis, which must be used in combination therapies. Such inhibitors could also help to distinguish the contribution of other PF‐3758309 targets, such as CDK7, to the high cellular activity of some PDAC models studied in this research. Our finding that a lipogenic PDAC subtype, as well as a TCA cycle signature, is linked to PF‐3758309 sensitivity could be useful for developing companion diagnostics to precisely use future AMPK inhibitors.

Importantly, our research uncovered that loss of AMPK can be compensated but triggers a concealed vulnerable cell state. To investigate whether AMPK inhibition is associated with specific vulnerabilities that can be exploited by AMPKi‐based combination therapies, we conducted a drug screening experiment in AMPK knock‐out PDAC cells. We observed that the ferroptosis inducer Erastin was more active in AMPK knock‐out PDAC cells. Consequently, a good synergy of Erastin and PF‐3758309 was observed in murine and human PDAC models. AMPK has been shown to be important for several layers of ferroptosis defense. For instance, it directly phosphorylates transcription factors that orchestrate anti‐oxidative programs, including members of the FoxO transcription factor family^[^ ^58^, ^59^ ^]^ as well as NRF2.^[^ ^60^ ^]^ Moreover, it blocks metabolic pathways that consume NADPH, including fatty acid synthesis through phosphorylation of ACC1,^[^ ^61^ ^]^ while maintaining continuous TCA flux^[^ ^47^, ^62^ ^]^ for NADPH production. Consistent with the role of AMPK in redox homeostasis, our RNA‐Seq analysis of genetic gain‐ and loss‐of‐function models demonstrated a connection of AMPK to gene sets related to glutathione metabolism. Lastly, our data are in line with recent observations, demonstrating that cancer cells with high AMPK activity are resistant to ferroptosis induction.^[^ ^63^ ^]^ Restraining the biosynthesis of polyunsaturated fatty acids (PUFA) by AMPK‐mediated ACC phosphorylation limits ferroptosis,^[^ ^63^ ^]^ which is a cell death that is dependent on lipid peroxidation and the abundance of PUFA.^[^ ^64^ ^]^

In summary, our data may point to a position of AMPK at the crossroads of metastatic phenotypes and the ferroptosis pathway in PDAC, opening new therapeutic options and research directions. Furthermore, we characterized a novel chemical scaffold for the development of specific AMPK inhibitors.

## 4 Experimental Section

### 2D Cell Culture

Cells were cultivated either in DMEM high glucose (#D5796, Sigma‐Aldrich, St. Louis, Missouri, USA) supplemented with 10% (v/v) FCS (#S0615, Sigma‐Aldrich) or in the case of primary human cell lines with 3:1 Keratinocyte‐SFM Medium (#17005042, ThermoFisher Scientific, Waltham, Massachusetts, USA) supplemented with 10% (v/v) FCS (#S0615, Sigma‐Aldrich), 0.5 mg mL^−1^ bovine pituitary extract (Sigma‐Aldrich) and 0.05 ng mL^−1^ hEGF (#E9644, Merck, Darmstadt, Germany) and RPMI‐1640 (#R8758, Sigma‐Aldrich) supplemented with 10% (v/v) FCS (#S0615, Sigma‐Aldrich) at 37 °C and 5% CO_2_. After reaching 80–90% confluency, cells were sub‐cultured. Specifically, the medium was removed, and cells were washed once with PBS (#20012019, ThermoFisher Scientific) before adding 0.05% EDTA (P10‐026100, PAN‐Biotech, Aidenbach, Germany) in PBS (#20012019, ThermoFisher Scientific) to detach the cells from the flask (Sarstedt, Nümbrecht, Germany). Cells were diluted in a pre‐warmed growth medium and the cell suspension was either recultured or used for subsequent experiments. All cell lines were confirmed to be mycoplasma‐free by a recently described PCR‐based detection protocol^[^ ^65^ ^]^ and cultivated for <30 passages.

### Plasmid Constructions

For lentiviral overexpressing construct, we employed pLenti PGK Puro plasmid (Addgene, #19068, RRID: Addgene_19068) as the backbone. Murine *Prkaa1* was amplified from cDNA and inserted into the pENTR vector (Addgene, #17398, RRID: Addgene_17398). Subsequently, Gateway assembly was employed to transfer the *Prkaa1* cDNA from pENTR to the pLenti PGK Puro vector. All the generated constructs were confirmed by Sanger sequencing (Eurofins Scientific, Luxemburg, Luxemburg). The final construct pLenti PGK puro *Prkaa1* was deposited at Addgene (Addgene, #204356). Primers used for the amplification of murine *Prkaa1* can be found in Table S1 (Supporting Information).

### Lentivirus Production and Transduction

For the production of lentiviral particles, HEK293FT cells (RRID: CVCL_6911) were seeded in 10 cm dishes in 10 mL DMEM high glucose (#D5796, Sigma‐Aldrich) with 10% (v/v) FCS (#S0615, Sigma‐Aldrich). The next day, a plasmid mix consisting of 1.25 µg psPax2 packaging plasmid (Addgene, #12260, RRID: Addgene_12260), 0.75 µg pMD2 VSV‐G envelope expressing plasmid (Addgene, #12259, RRID: Addgene_12259) and 2 µg lentiviral vector expressing the gene of interest was prepared and mixed with 270 µL Opti‐MEM I Reduced Serum Media (#31985062, ThermoFisher Scientific). Next, 18 µL TransIT‐LT1 (Mirus Bioscience, Madison, USA) were added, mixed by pipetting, and incubated for 20–30 min at room temperature to allow the transfection complex formation. Afterward, the mixture was added to the HEK293FT cells (RRID: CVCL_6911) and incubated overnight at 37 °C. On day 1 post‐transfection, the medium was changed to 4 mL DMEM high glucose (#D5796, Sigma‐Aldrich) with 30% FCS (#S0615, Sigma‐Aldrich). Lentivirus supernatant was collected two and three days later, pooled, filtered through a 0.2 µm filter, and stored at −80 °C until further use.

For lentiviral transduction, 100 000 cells were seeded in a 6‐well plate (#83.3920, Sarstedt). The next day, the media was replaced with 1 mL of the lentivirus‐containing medium with 8 µg mL^−1^ Polybrene (#TR‐1003, Sigma‐Aldrich). After 8 h, 1 mL of culture medium was added. After 24 h, the medium was changed to culture medium. After an additional 24 h, transduced cells were selected with 8 µg mL^−1^ Puromycin for 5 days until all cells in the control well were eliminated.

### Growth Curves

To determine the growth rate of cell lines, 1000 cells per well were seeded in 100 µL of growth medium in at least technical triplicates in white 96 well plates (#137101, ThermoFisher Scientific). After 24‐h intervals, 25 µL of CellTiter‐Glo Reagent (#G7570, Promega, Madison, Wisconsin, USA) prepared according to the manufacturer's instructions was added to each well and incubated for 20 min on an orbital shaker protected from light. Luminescence was measured on a microplate reader (VICTOR X4 2030‐0040, PerkinElmer Cellular Technologies Germany GmbH). Experiments were performed in technical triplicates and at least in biological triplicates unless otherwise stated.

### Pharmacotyping of 2D Cell Lines

For pharmacotyping of 2D cell lines, 1000 cells per well were seeded in white 96 well plates (#137101, ThermoFisher Scientific) in 100 µL of growth medium. After 24 h of incubation at 37 °C and 5% CO_2_, cells were treated with 20 µL of drug dilution in growth media per well. After a further 72 h of incubation at 37 °C and 5% CO_2_, 25 µL of CellTiter‐Glo Reagent (#G7570, Promega) prepared according to the manufacturer's instructions was added to each well and incubated for 20 min on an orbital shaker protected from light. Luminescence was measured on a microplate reader (VICTOR X4 2030‐0040). Experiments were performed in technical triplicates and at least in biological triplicates unless otherwise stated.

### 3D Cell Culture

The primary patient‐derived PDAC organoids were isolated as recently described^[^ ^65^ ^]^ or according to a published protocol.^[^ ^66^ ^]^ To cultivate the primary patient‐derived PDAC organoids, cells were resuspended in Matrigel Growth Factor Reduced (GFR) Basement Membrane Matrix‐ Phenol Red‐free – LDEV‐free (#356231, Corning) in 24‐well plates. Cultivation medium was added after Matrigel solidification. PDO media consisted of Advanced DMEM/F‐12 medium (#11540446, Gibco), supplemented with 10 nm HEPES (#11560496, Gibco), 1x‐GlutaMAX (#11574466, Gibco), 0.1% BSA (#A7030, Sigma‐Aldrich), 10% R‐spondin1‐Conditioned medium (R‐spondin1‐Conditioned medium overexpressing cell line HEK293T), 1x‐B27 (Thermo‐Fischer), 10 nM Nicotinamide (Sigma‐Aldrich), 1.25 mM N‐ Acetylcysteine (#17504044, Sigma‐Aldrich), 100 µg mL^−1^ Primocin (#ant‐pm‐05, Invivogen), 100 ng mL^−1^ mNoggin (#120‐10C, Peprotech), 100 ng mL^−1^ hFGF10 (#100‐26, Peprotech), 10 nM hGastrin I (#10047‐33‐3, Tocris), 500 nM A83‐01 (#2939, Tocris), 10.5 µM Y‐27632 (#HY‐10583G, Hycultec). PDOs were maintained at 37 °C in 5% CO2.

### Pharmacotyping and Life Cell Imaging of 3D Cell Lines

The pharmacotyping experiments of PDOs B211, B203, B169, B226, B188, and B250 were performed as described.^[^ ^65^ ^]^ Treatment and analysis of lines PDO‐51T, PDO‐70T, and PDO‐74T were done as briefly described. Organoids were digested to a single‐cell suspension using TrypLE Express Enzyme (#12605028, Gibco). 1250 cells per well were mixed in a total volume of 50 µL per well containing 10% of Cultrex Reduced Growth Factor Basement Membrane Extract, Type 2 (#3536‐005‐02, R&Dsystems) and seeded into 384‐well white plates (#762827, Greiner) for cell viability assays or into 384‐well clear plates (#6236585, Greiner) for live cell imaging (Incucyte SX5, Sartorius). After 24 h, cells were treated with the indicated drug and incubated at 37 °C in 5% CO_2._ For viability assay, cells were cultured for 72 h, and cell viability was measured by adding 15 µL of CellTiter‐Glo Luminescent Assay (#G7573, Promega). Luminescence was measured using a VICTOR X4 2030‐0040 Multilabel Plate Reader. Clear plates were incubated for 7 days without media exchange and imaged using the Incucyte SX5 Live‐Cell Analysis System (Sartorius). Data were normalized to DMSO and analyzed using Incucyte Organoid Analysis Software and GraphPad Prism 9.

### Floating Collagen Gel Assay

Floating Collagen Gel Assays were performed as previously described.^[^ ^39^ ^]^ Briefly, 9091 LacZ control cells and corresponding *Prkaa1* KO1 and *Prkaa1* KO2 cells were seeded in a limiting dilution up to 20 cells per gel and cultured for 13 days.

Media changes were performed first after 72 h and then every 48 h until day 13. For every cell line, three technical replicates of 4 gels each were generated. In total, 253 organoids were imaged (LacZ: 63, KO1: 105, KO2: 85) and classified into their respective phenotypes.

### Organoid Branching

Two thousand cells were initially seeded as hanging drops in standard growth medium and allowed to incubate for 24 h. Subsequently, a 3D matrix comprising Matrigel (#356231, Corning) and Collagen I (#354231, Corning) in a 7:3 ratio was prepared as described in.^[^ ^67^ ^]^ The hanging drops were then pelleted and transferred into 30 µL of the 3D matrix, which was plated onto pre‐warmed 24‐well plates. After solidification of the droplets, each well was supplemented with 650 µL of pre‐warmed Advanced DMEM/F‐12 medium (Gibco), supplemented with 1% P/S and 1x ITS (#I3146, Merck) and 2.5 nM FGF2 (#CB‐1102024, PAN‐Biotech). Subsequent imaging of the organoids was performed on days 0, 1, and 2 using an IX83 Olympus microscope. Analysis of organoid outgrowth was conducted utilizing cellSens software (RRID: SCR_014551).

### Clonogenic Assay

Cells were seeded (density of 1–2 × 10^3^ cells per well, depending on growth rate) in 0.5 mL medium in 24 well plates (#83.3922.005, Sarstedt), and after 24 h, 0.5 mL inhibitor dilution was added. When the control wells were 80–90% confluent (≈ 7–10 days after seeding), the medium was removed and the cells were washed with PBS (#20012019, ThermoFisher Scientific) twice followed by the addition of 200 µL of 0.2% crystal violet (#T123, Roth, Karlsruhe, Germany) solution (2% (v/v) Ethanol (#2212, CHEMSOLUTE) in ddH₂O) and incubation on a shaker for 10 min at room temperature. Then the plates were washed with ddH₂O until clean, air‐dried, and scanned for visualization. For quantification, 600 µL 1% SDS (#CN30, Roth) was added to each well and the plate was incubated on a shaker until the stain was completely solubilized. Absorbance measurements were performed at 570 nm in a photo spectrometer (Multiskan FC, ThermoFisher Scientific).

### Synergy Estimation

Clonogenic assay data were used to calculate the synergy scores with the online software Synergy Finder (v1.0).^[^ ^68^ ^]^

### Drug Screening

The cherry‐picked compound libraries were purchased from Selleck Chemicals (Houston, USA) (Tables S2,S3, Supporting Information). The compound library was diluted in 384 well plates (#3765, ThermoFisher Scientific) in DMSO in 7 concentrations of each compound to attain the following final treatment concentrations: 10, 3.3, 1.1, 0.37, 0.12, 0.04, and 0.014 µm and DMSO as control. The optimal cell number for the screen was determined to ensure growth in the log phase at the end‐point measurement. For each screen, cells were seeded in white 96 well plates (#137101, ThermoFisher Scientific) in 100 µL culture medium using a Multidrop Combi dispenser (ThermoFisher Scientific). The screening was conducted as one biological replicate performed as technical duplicates. After 24 h of incubation, cells were treated with the diluted compound library using a liquid handling manual pin tool (V&P Scientific, San Diego, California, USA). Cell viability was also measured 24 h after seeding, and doubling times in an hour were calculated by dividing cell viability at the endpoint by the cell viability 24 h after seeding. Cell viability was measured using the CellTiter‐Glo Luminescent Assay (#G7573, Promega) as described above.

Dose‐response curves were generated using the R package GRmetrics.^[^ ^69^, ^70^ ^]^ Half maximal inhibitory concentration (IC_50_) and area under the curve (AUC) were used as drug sensitivity measures. For the gain‐ and loss‐of‐function drug screening experiments, the fold change of the IC_50_ (FC(IC_50_)) and delta AUC (ΔAUC) was calculated, and drugs were ranked according to these measures. Drug sensitivity parameters are summarized in Tables S4–S6 (Supporting Information).

### Glycolysis Stress Test

XF96e Extracellular Flux Analyzer (Agilent Technologies, Santa Clara, California, USA) was used to evaluate the rate of extracellular acidification in cells. The manufacturer's instructions for the Seahorse XF Glycolysis Stress Test Kit User Guide (#103020‐400, Agilent Technologies) were followed when completing the assay. For this assay, 35 000 cells were plated on a Seahorse Plate (#101085‐004, Agilent). The next day, XF DMEM Buffer (supplemented with 1 mM pyruvate and 2 mM glutamine) was used to test the media's initial acidification, and subsequent extracellular acidification rate (ECAR) measurements were taken after the addition of 10 mM glucose, 3 µM Oligomycin, and 50 mM 2‐deoxy‐D‐glucose.

### Mito Stress Test

According to instructions provided by the manufacturer in the Seahorse XF Cell Mito Stress Test Kit User Guide (#103016‐400, Agilent Technologies), the Seahorse XF96e Extracellular Flux Analyzer was used to measure the oxygen consumption rate (OCR) in cells. For the analysis, 35 000 cells were plated onto a Seahorse Plate (#101085‐004, Agilent). The next day, XF DMEM buffer (supplemented with 1 mM pyruvate, 2 mM glutamine, and 10 mM glucose) was used to test baseline respiration. Following the addition of 3 µM Oligomycin, 1.5 µM CCCP, and 0.5 µM Antimycin/Rotenone, OCR was further measured under varying metabolic conditions.

### Caspase 3/7 Assay

To evaluate apoptosis, 1000 cells per well were seeded in 100 µL of growth medium in a white 96‐well plate (#137101, ThermoFisher Scientific). After 48 h, the caspase 3/7 assay (#G8091, Promega) was used according to the instructions provided by the manufacturer. Experiments were performed as two technical replicates and three biological replicates.

### GSH/GSSG‐Glo Assay

To detect and quantify total glutathione ratios, 1000 cells per well were seeded in 100 µL of growth medium (DMEM supplemented with 10% FCS) in a white 96‐well plate (#137101, ThermoFisher Scientific). After 24 h, cells were treated with 20 µL of the indicated drug and incubated at 37 °C in 5% CO_2_ for the indicated period. Total glutathione ratios were measured using GSH/GSSG‐Glo Assay (#V6611, Promega) according to the manufacturer's instructions.

### GEPIA Analysis

For Gene Expression Profiling Interactive Analysis (GEPIA) (RRID: SCR_018294)^[^ ^71^ ^]^ the box plot function was used. The curated PAAD dataset with 151 samples was matched to 171 GTEx samples (https://www.cancer.gov/tcga).^[^ ^72^ ^]^ The following parameters were used for analysis: Log_2_FC cutoff: 0.58, *p*‐value cutoff: 0.05. For statistical analysis, a one‐way ANOVA was performed.

### mRNA Isolation

For RNASeq, cells were seeded in 6‐well plates. Cell numbers were adjusted to their growth rate. mRNA extraction was performed on ice. The growth medium of cultured cells was discarded, and cells were washed twice with 500 µL PBS. Cell extracts were obtained using 300 µL of 1x RLT buffer containing (#79216, Qiagen, Hilden, Germany) 1:100 β‐Mercaptoethanol (#M6250, Sigma‐Aldrich) per well of a 6‐well plate. Cells were scraped from the plate, transferred to 1.5 mL tubes, and isolated using the Maxwell 16 LEV simply RNA Tissue Kit (#AS1270, Promega), following the manufacturer's instructions. RNA concentration was measured using a Nandrop spectrophotometer (Peqlab Biotechnologie GmbH, Erlangen, Germany) and samples were stored at −80 °C.

### RNA Reverse Transcription

cDNA synthesis was performed using the TaqMan reverse transcription buffer (#N8080234, Thermo Scientific), following the manufacturer's instructions. 2 µg RNA was used to generate 100 µL cDNA and samples were stored at −80 °C until further use.

### Quantitative Real‐Time PCR

Primers for quantitative PCR are depicted in Table S1 (Supporting Information) and were obtained from Eurofins Scientific. Primer efficiency was tested and ranged from 85–115%. 100 nM of the primer and SYBR Green Master Mix (#4309155, Thermo Scientific) were used according to the manufacturer's instructions for quantitative mRNA analysis using a real‐time PCR analysis system with the following cycling conditions: 95 °C 10 min, 40 x (95 °C 15 s, 60 °C 1 min), 95 °C 15 s, 60 °C 1 min, 95 °C 15 s. All samples were normalized to β‐actin. Data analysis was performed using the StepOne Software v2.3 (RRID:SCR_014281, Life Technologies Corporation) according to 1/ΔCT / 2^(−ΔCt) method.^[^ ^73^ ^]^

### Bulk RNA‐Seq

RNA‐Seq was performed at the Sequencing Core Unit at the TranslaTUM, Technical University Munich (TUM) or the NGS Integrative Genomics Core Unit, University Medical Center Göttingen (UMG). For the RNASeq performed at the Sequencing Core Unit at the TranslaTUM, library preparation for bulk‐sequencing of poly(A)‐RNA was done as previously described.^[^ ^74^ ^]^ Subsequent steps were performed as previously published.^[^ ^75^ ^]^ For the RNASeq performed at the NGS Integrative Genomics Core Unit, University Medical Center Göttingen (UMG), sequence images were transformed with Illumina software BaseCaller to BCL files, which was demultiplexed to fastq files with bcl2fastq v2.20. The sequencing quality was asserted using FastQC (http://www.bioinformatics.babraham.ac.uk/projects/fastqc/). Sequences were aligned to the reference genome Homo sapiens (GRCh38.p13, https://www.ensembl.org/Homo_sapiens/Info/Index) using the STAR RNA‐Seq alignment tool^[^ ^76^ ^]^ (version 2.7.8a) allowing for 2 mismatches within 50 bases. Subsequently, read counting was performed using featureCounts.^[^ ^77^ ^]^ Differential gene expression analysis was performed with R‐Studio (R version 4.0.2 (2020‐06‐22), open‐source license) and DEseq2. Genes with “sum(read counts) < n(sequenced samples)” were removed and the remaining counts were normalized and transformed using regularized log_2_ transformation (rlog) implemented in the DEseq2 package. RNAseq data can be accessed via the European Nucleotide Archive (ENA) (PRJEB63203).

### Cluster Analysis

Analysis of RNA‐Seq of murine Kras^G12D^‐driven cell lines (n = 38) was based on a recently published transcriptome dataset and corresponding annotations.^[^ ^27^ ^]^ Information for metastasis formation (No, Yes), the grading of the respective tumors (Undifferentiated, G3, G2, G1), and murine PDAC clusters (mClusters) (C1, C2a, C2b, C2c, outlier) were derived from.^[^ ^27^ ^]^ The cellular morphology (mesenchymal, epithelial) of the cell lines was determined by microscopic investigation. AMPK subunits were hierarchically clustered (method: average, distance: euclidean) and the resulting cluster tree was stratified into three main clusters. For metabolic subtyping, we performed hierarchical clustering on the differentially expressed genes specific to the lipogenic and glycolytic PDAC subtypes sourced from Daemen et al.^[^ ^36^ ^]^ using predefined criteria (log_2_(FC)>1 or ←1, p‐adj<0.05). The resulting cluster tree was stratified into the two main clusters aligning with the described glycolytic and lipogenic subtypes.

### DepMap Portal

Protein array and metastatic potential (MetMap 500: all5) data filtered by “Pancreas” were used for the Custom Analyses (Type of analysis: Pearson correlation) available in the Cancer Dependency Map Portal (RRID:SCR_017655). For the validation of the observed correlations, proteomic and metastatic potential (MetMap 500: all5) data filtered by “Pancreas” was downloaded from the DepMap portal and correlated to the AMPK subunits by using the cor(method = “pearson”) and cor. test(method = “pearson”) function in R‐Studio (R version 4.0.2 (2020‐06‐22), open‐source license).

### GSEA

For gene set enrichment analysis (GSEA) between groups, the open‐source tool GSEA v4.3.2 or Genetrail 3.2^[^ ^78^ ^]^ (RRID:SCR_006250) was used. For single sample GSEA (ssGSEA), the R package GSVA^[^ ^79^ ^]^ was used. Genesets HALLMARK and KEGG were downloaded from the MSigDB homepage (RRID: SCR_016863).

### scRNA‐Seq Analysis

Single‐cell nuclear transcriptomic data of 43 primary PDAC tumor specimens was downloaded from GSE202051.^[^ ^24^ ^]^ H5ad files were converted to H5seurat files with R (V. 4.2.2) and Rstudio (V. 2023.3 using the packages Seurat (V. 4.3.0) and SeuratDisk (V. 0.0.0.9020). Subsequent analyses were performed with package Seurat (V. 4.3.0). Cell subtypes were filtered for untreated cells according to the provided annotations using the subtype function of Seurat. Additionally, the percentage of expressed mitochondrial genes was determined using PercentageFeatureSet(data pattern = “^MT‐”), and cells with mitochondrial genes >5% were filtered out. Principle component analysis (PCA) was performed using the subsetted data with npcs = 40. Subsequently, UMAP was performed with dims = 1:20 and reduction “pca”. Visualization of single cells and *PRKAA1* expression density was performed using the additional packages Nebulosa (V. 1.8) and viridis (V.0.6.2) and the functions DimPlot, plot_density (reduction = “umap” and provided annotations) as well as DotPlot.

### Protein Extraction

Protein extraction was performed on ice. The growth medium of cultured cells was discarded, and cells were washed twice with PBS. Protein lysates were obtained using 100 µL of 1x RIPA buffer (150 mM NaCl, 10 mM TRIS, 0.1% (w/v) Sodiumdeoxychelate, 0.1% (w/v) SDS, 1% (v/v) NPO₄) containing 1x protease‐inhibitor (#4693132001, Roche, Basel, Switzerland) as well as 1x phosphatase inhibitor (#4906837001, Roche) per 10 cm cell culture dish. Cells were scraped from the plate, transferred to 1.5 mL tubes, and centrifuged for 15 min at 4 °C and 16 000 x g, and the supernatant was stored at −80 °C.

### Bradford Assay

The protein concentration of cell extracts was estimated using Bradford reagent (Sigma‐Aldrich). Absorbance measurements were performed at 595 nm in a photo spectrometer (Multiskan FC, ThermoFisher Scientific) and subsequently, protein extracts were adjusted to desired protein concentrations in 5x LaemmLi buffer (250 mm Tris‐HCl (pH 6.8), 4% (w/v) SDS, 40% (v/v) Glycerol, 0.05% (w/v) Bromphenolblue, 5% (v/v) β‐Mercaptoethanol). After boiling the samples for 5 min at 95 °C, samples were stored at −20 °C.

### SDS‐PAGE and Western Blotting

For sodium dodecyl sulfate‐polyacrylamide gel electrophoresis (SDS‐PAGE), the Western Blot System Mini‐PROTEAN Tetra System (Bio‐Rad, Hercules, California, USA) was used. Depending on the protein size, 7.5–12% gels were prepared. The gels were run with 1x running buffer (192 mM Glycine, 25 mM TRIS, 3.47 mM SDS) for 2–3 h at 80–120 V. For Western blotting, gels were transferred onto nitrocellulose membranes in 1x transfer buffer (192 mM Glycine, 25 mM TRIS, 20% (v/v) Methanol) for 2 h at 350 mA. To minimize unspecific antibody binding, membranes were blocked with 5% skim milk in 1x TBS and subsequently incubated with a primary antibody overnight at 4 °C. Prior to incubation with a secondary antibody for 2 h at room temperature, the membranes were washed with 0.1% Tween in 1x TBS 3 times for 15 min. After incubation with the secondary antibody, the washing steps were repeated as described before. Depending on the fluorescent secondary antibody used, membranes were scanned with Odyssey Fc (LI‐COR, Lincoln, Nebraska, USA) at 488, 700, or 800 nm to visualize specific protein bands. To detect chemiluminescent secondary antibodies, membranes were incubated in HRP substrate for 10 s before scanning with ChemiDoc MP (Bio‐Rad). For quantification, Image Studio Light version 5.2 software was used. For phosphorylation level analysis, the same lysates were transferred to two separate membranes and incubated either with phospho‐ or pan‐antibodies. First, phospho‐ and pan‐bands were normalized to their respective loading control. Subsequently, the relative phosphorylation levels were calculated. Antibodies and dilutions can be found in Table S7 (Supporting Information).

### Kinobeads Assay

Dose‐dependent competition pulldown assays using kinobeads ε were performed as previously described.^[^ ^80^ ^]^ Deviations from the protocol were the use of 2.5 mg protein of cell lysate per pulldown experiment, and the use of the following compound concentrations: 0.3, 1, 3, 10, 30, 100, 300, 1000, 3000, and 30 000 nM, or vehicle.

LC‐MS/MS measurement was carried out on a micro‐flow LC system built by combining a modified Vanquish pump with the autosampler of the Dionex UltiMate 3000 nano HPLC System (Thermo Scientific) coupled to an Orbitrap Fusion Lumos Tribrid instrument (Thermo Scientific). Dried peptides were reconstituted in 0.1% formic acid and loaded directly onto an Acclaim PepMap 100 C18 column (2 µm particle size, 1 mm ID × 150 mm, Thermo Scientific) heated at 55 °C. Samples were separated using a 15‐min linear gradient of 7–32% solvent B (solvent A: 0.1% formic acid, 3% DMSO in HPLC grade water; solvent B: 0.1% formic acid, 3% DMSO in ACN) at a flow rate of 50 µL min^−1^. Peptides were ionized using an electrospray voltage of 3.5 kV, a capillary temperature of 325  °C, and a vaporizer temperature of 125  °C. Sheath, aux, and sweep gas were used at a flow rate of 32, 5, and 0, respectively. MS1‐spectra were acquired in the orbitrap at a resolution of 120 000 using a maximum injection time of 50 ms and an AGC target value of 4 × 10e5. Ions were fragmentation by HCD with a normalized collision energy of 35. MS2‐spectra were acquired in the linear ion trap in rapid scan mode using a maximum injection time of 10 ms and an AGC target value of 1×10e4. The cycle time was 0.6s, with isolation windows of 0.4 m/z and dynamic exclusion of 12 s.

Raw files were searched against the UniProtKB Mouse Reference Proteome database (UP000000589, downloaded on April 20th, 2022) using MaxQuant (v1.6.12.0), with labelfree quantification (LFQ) and “match‐between‐runs” enabled. The results were filtered for potential contaminants, reversed hits, and proteins identified only by PTMs. For data analysis, LFQ intensities were normalized to vehicle control to retrieve residual binding at each drug dose. The resulting ratios were fitted to a four‐parameter log‐logistic regression model using the “drc” package in R to retrieve curve parameters.

### Docking Analysis

For the docking study, the structures of AMPKα, CDK7, and PAK4 (Table S8, Supporting Information) were downloaded from the Protein Databank (http://www.rcsb.org). The inhibitor PF‐3758309 has been cocrystallized with PAK4 (PDB ID 2 × 4Z) and was used to test the docking method. The following steps of protein preparation were executed using the graphical user interface of Maestro (RRID: SCR_016748). Subsequently, Schrödinger's Protein Preparation Wizard was used to prepare the protein structures for ligand docking by adding hydrogen atoms, filling in missing side chains, capping the chains'termini, and optimizing the hydrogen bond network (at pH 7.4) (RRID: SCR_016745^[^ ^81^ ^]^). Finally, an energy minimization step was executed using OPLS 2005 as a force field.^[^ ^82^, ^83^, ^84^, ^85^ ^]^ The prepared structure was solvated with the aid of TIP3P water molecules and neutralized with chloride ions in an orthorhombic box with a margin of 10 Å to the protein surface. Desmond was used afterward in order to perform an additional energy minimization step in the presence of water^[^ ^86^ ^]^ (RRID: SCR_014575). The minimized protein‐ligand complex served as a template for generating the receptor grid assigning the cocrystallized inhibitor as the center of the grid. All inhibitor structures for docking were prepared using Schrödinger's Ligprep (RRID: SCR_016746) in standard settings including Epik (RRID: SCR_016745^[^ ^86^, ^87^ ^]^) for the generation of ionization states and utilizing the OPLS 2005 force field. Confgen was used afterward to generate 64 diverse conformers per inhibitor (RRID: SCR_023928^[^ ^88^ ^]^). These conformers served as an input for the subsequent docking procedure for which Schrödinger's Glide was used in Standard Precision (SP) mode (RRID: SCR_000187).^[^ ^89^, ^90^ ^]^ The resulting binding poses were visualized using PyMOL (RRID: SCR_000305) and MOE 2019.01 (RRID: SCR_014882). The described docking setup was first tested with the cocrystallized inhibitors (PF‐3758309 in PAK4, related pyrazolo[1,5‐a]pyrimidines in CDK7, and Staurosporine in AMPKα) to check whether RMSD values below 1.2 Å can be reproduced (Table S8, Supporting Information).

### Immunohistochemistry

Immunohistochemistry (IHC) was performed using a Bond RXm system (Leica, Wetzlar, Germany, all reagents from Leica) with a primary antibody against AMPKα (RRID: AB_722764, Clone Y365, Dilution 1:400) as well as P‐AMPKα (RRID: AB_331250, Clone Thr172, Dilution: 1:100). Briefly, slides were deparaffinized using deparaffinization solution. For AMPKα the tissue samples were pretreated with Epitope retrieval solution 1 (corresponding to citrate buffer pH 6) for 30 min, and for p‐AMPKα Epitope retrieval solution 2 (corresponding to EDTA buffer pH 8) was applied for 30 min. Antibody binding was detected with a polymer refine detection kit (#DS9800, Leica) without post‐primary reagent and visualized with DAB as a dark brown precipitate. Counterstaining was done with hematoxyline.

IHC staining for AMPKα and p‐AMPKα was performed on PDAC Tissue Microarrays (TMA) of 107 patients (cohort previously described^[^ ^91^, ^93^ ^]^). The Ethics committee of Charite University approved the use of this cohort for biomarker investigation (EA1/06/2004). The IHC slides were evaluated in relation to the intensity of the staining reaction and the proportion of positive tumor cells. Additionally, to combine intensity and the proportion of positive tumor cells both were multiplied, whereas low intensity equaled 1, high intensity respectively 3, resulting in a combined score from 0 to 3. Staining intensities were further divided into three levels using Cutoff Finder.^[^ ^92^ ^]^ We used the following values for AMPK: 0, <1.56, >1.56, and for P‐AMPK: 0, <1.46, >1.46.

Subtyping of the human PDAC cohort was performed based on a previously published IHC surrogate marker approach^[^ ^91^, ^93^ ^]^ with KRT81 positive neoplasms mainly corresponding to the quasi‐mesenchymal/squamous/basal‐like subtype, HNF1A positive tumors predominantly reflecting an exocrine‐/ADEX‐like type of neoplasms and double‐negatives primarily corresponding to a classical subtype as characterized on the transcriptomic level by different authors.^[^ ^22^ ^]^

### Statistical Analysis

Graphic depictions and statistical analysis were generated using Graph Pad Prism 9, R v4.3, and GSEA v4.3.2. Data are either presented as mean ± standard deviation (SD) or as truncated violin plots with median (—) and quartiles (^…^). Outliers are defined as >2 SD ± mean. Statistical analysis as well as pre‐processing for each experiment is described either in the method section and/or in the figure legends. All data were obtained from at least three independent experiments unless otherwise stated. Specific numbers of technical and biological replicates for each experiment are stated in the figure legends. The resulting p‐values are indicated in the respective figures. Notably, *p*‐values below 0.05 are denoted with one star (*), those below 0.01 with two stars (**), and those below 0.001 with three stars (***). Additionally, if a *p*‐value falls between 0.1 and 0.05, the exact value is explicitly stated. A comparison was considered significant if the p‐value was equal to or below 0.05. To test directional hypotheses, we used one‐tailed *t*‐tests. In cases where multiple statistical tests were performed on the same dataset, a Bonferroni or Tukey correction for multiple testing was applied and indicated in the figure legends

### Schematic Drawings

Schematic drawings were generated with the support of Inkscape (http://www.inkscape.org/).

### AI‐Assisted Technologies in the Writing Process

During the preparation of this work, the author(s) used Grammarly and large language models in order to improve language and readability. After using these tools, the author(s) reviewed and edited the content as needed and take(s) full responsibility for the content of the publication.

### Ethics Approval Statement and Patient Consent Statement

The primary human PDAC models were established and analyzed in accordance with the declaration of Helsinki and were approved by the local ethical committee of University Medical Center Göttingen (UMG) (vote 11/5/17) and the Technical University Munich, Klinikum rechts der Isar (Project 207/15). Written informed consent from the patients for research use was obtained prior to the investigation. The Ethics committee of Charite University approved the use of the PDAC cohort for biomarker investigation (EA1/06/2004).

## Conflict of Interest

The authors declare no conflict of interest.

## Author Contributions

C.S., J.H., and F.G. contributed equally to this work. All data and the work reported in the paper have been performed or were generated by the authors unless specified in the text. C.S., J.H., F.G., M.R., and G.S. performed conception and design of the study; C.S., J.H., F.G., S.H., L.K., F.S., C.T.C., S.J., A.P., F.W., T.R., A.M.A., R.Ö., A.B., D.M., M.W., K.S., W.S., G.S. performed acquisition of data and/or analysis, curation, and interpretation of data; C.S., J.H., F.G., F.H., S.J., A.P., F.W., C.T.C., A.M.A., C.F., C.S., R.Ö., P.R., D.M., P.S., V.E., L.C., E.H., M.G., M.G., M.W., K.S., R.R., B.K., W.S., M.R., D.S., G.S. performed generation of important models and contribution of essential resources, technology, and acquired funding; C.S., G.S. performed drafting of the manuscript. All authors did revisions for important intellectual content and approved the final version for publication.

## Supporting information

Supporting Information

Supporting Table

## Data Availability

The data that support the findings of this study are available from the corresponding author upon reasonable request.
