# Supplementary material for: A Novel AMPK Inhibitor Sensitizes Pancreatic Cancer Cells to Ferroptosis Induction
Source: Adv Sci (Weinh). 2024 Jun 17;11(31):2307695. doi: 10.1002/advs.202307695 (PMC11336956; doi:10.1002/advs.202307695)
Supplement: Supplementary file 1 — Supporting Information [file ADVS-11-2307695-s002.docx]

**
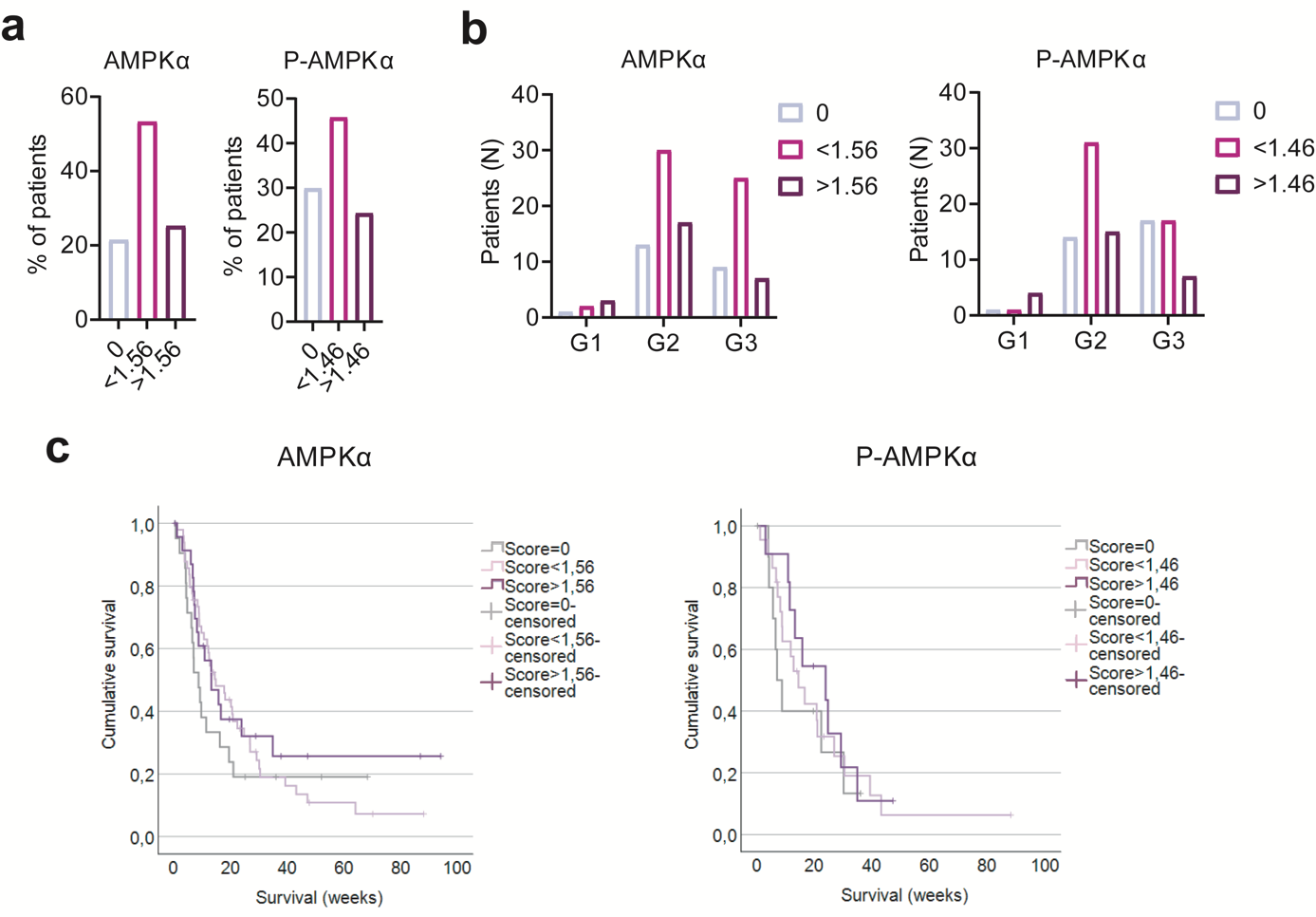
**

**Figure S1 | AMPKα in pancreatic cancer.** Staining intensities of P-AMPKα and AMPKα in PDAC (N = 107). Staining intensities are divided into three levels using Cutoff finder. **a,** Percentages of PDACs with no, low or high expression (left panel) / phosphorylation (right panel) of AMPK. **b,** The number of patients with specific staining intensity is shown. P-AMPKα (right panel) and AMPKα (left panel) stained pancreatic cancers classified as G1 (N = 6), G2 (N = 60), G3 (N = 41). Statistical analysis was performed by chi-squared test. **c,** Cumulative survival of pancreatic cancer patients based on staining intensities of P-AMPKα (right panel) and AMPKα (left panel). Staining intensities are divided into three levels using Cutoff finder (AMPKα: 0,<1.56,>1.56; P-AMPKα: 0,<1.46,>1.46).

**
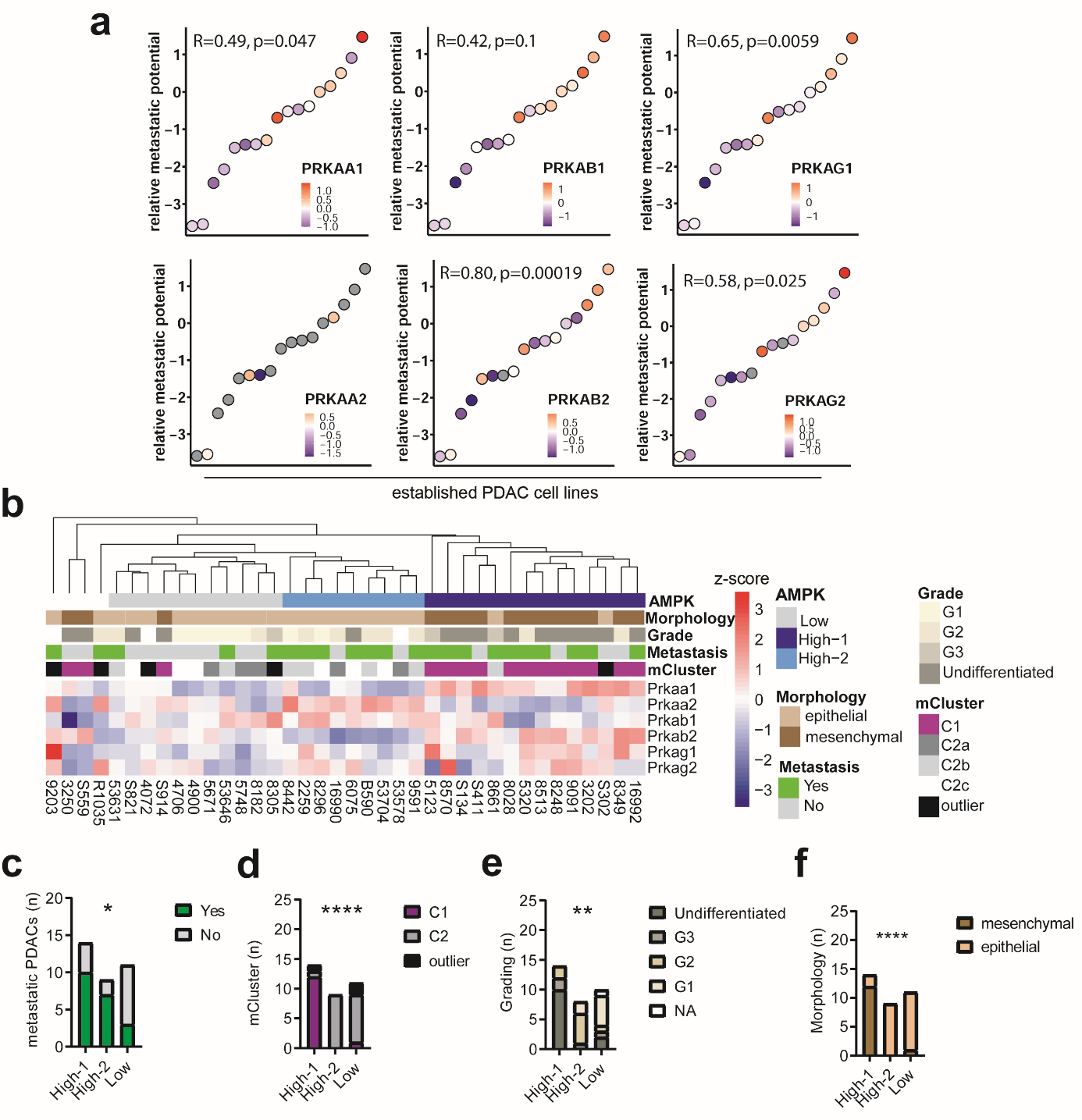
**

**Figure S2 | Prkaa1 is associated with a metastatic PDAC phenotype.** **a,** Data was accessed via https://depmap.org**.** Correlation of AMPK subunit expression with metastatic potential in human PDAC cell lines. PDAC cell lines are ordered by their relative metastatic potential. Relative AMPK subunit expression color-coded. Pearson correlation coefficient (R) and p-value (p) are shown. **b,** Heatmap of AMPK subunit expression in murine Kras^G12D^-driven cell lines (N = 38). Subunit expression was determined using RNA-Seq. Annotations are given for AMPK cluster (Low, High-2, High-1), metastasis formation (No, Yes) the cellular morphology of the cell lines, the grading of the respective tumors (Undifferentiated, G3, G2, G1) and their annotated murine PDAC cluster (mCluster) (C1, C2a, C2b, C2c, outlier). Clustering method: average, clustering distance: euclidean. **c,** Quantification of metastatic PDACs in AMPK clusters. Statistical analysis was performed by chi-squared test. **d,** Quantification of mClusters in AMPK clusters. Statistical analysis was performed by chi-squared test. **e,** Quantification of grading of the respective tumors in AMPK clusters. Statistical analysis was performed by chi-squared test. **f,** Quantification of the cellular morphology in AMPK clusters. Statistical analysis was performed by chi-squared test. *:p-value<0.05, **:p-value<0.01, ****:p-value<0.0001.

**
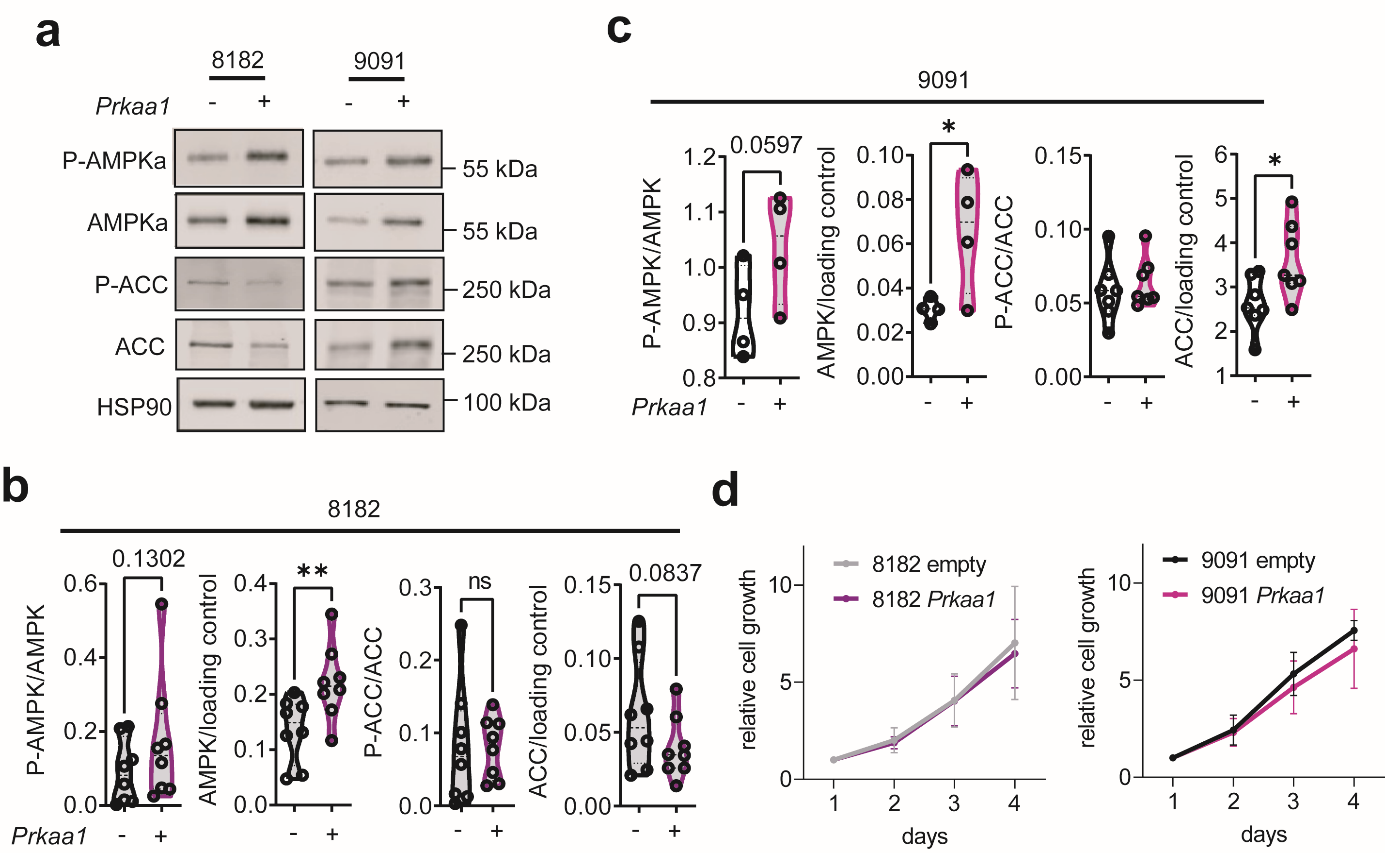
**

**Figure S3 | Characterization of PRKAA1 overexpression in murine PDAC cells. a,** Western blots of AMPK pathway in empty vector control (-) and Prkaa1 (+) overexpressing murine PDAC cells. AMPK pathway was investigated using P-AMPKα, AMPKα, P-ACC, and ACC antibodies. Hsp90 was used as loading control. Same lysates were transferred to two membranes and subsequently, incubated with either pan or phospho-antibodies and used to determine relative phosphorylation level of protein of interest. **b,** and **c,** Quantification of **a,**. Statistical analysis was performed by a one-tailed unpaired t-test. **d,** Growth curves of empty vector control (-) and Prkaa1 (+) overexpressing PDAC cells. 1,000 cells were seeded in 96-well plates and viability was measured each day for four subsequent days. Relative cell growth to day one is plotted on y-axis. ACC: Acetyl-CoA carboxylase, Hsp90: Heat shock protein 90, P-: Phosphorylation, ns: not significant, *: p<0.05, **: p<0.01.


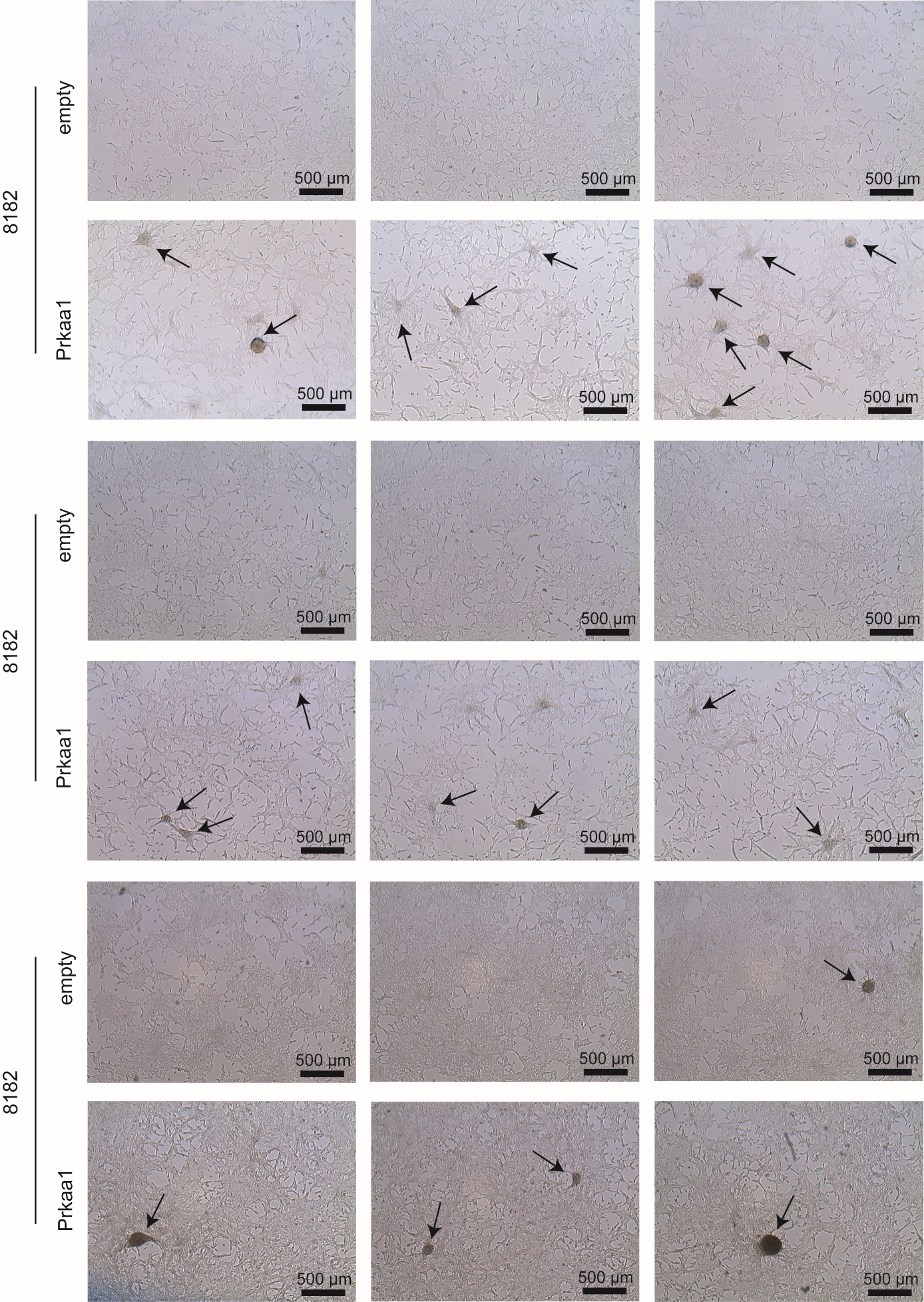


**Figure S4 | Microscopic pictures of 8182 cells with *Prkaa1* overexpression.** 8182 control (empty) and Prkaa1 overexpressing cells were cultured on 10 cm dishes for 3 days and pictures were taken using 10 x magnification. Scalebar corresponds to 500 µm. Three biological replicates with three pictures each are shown.


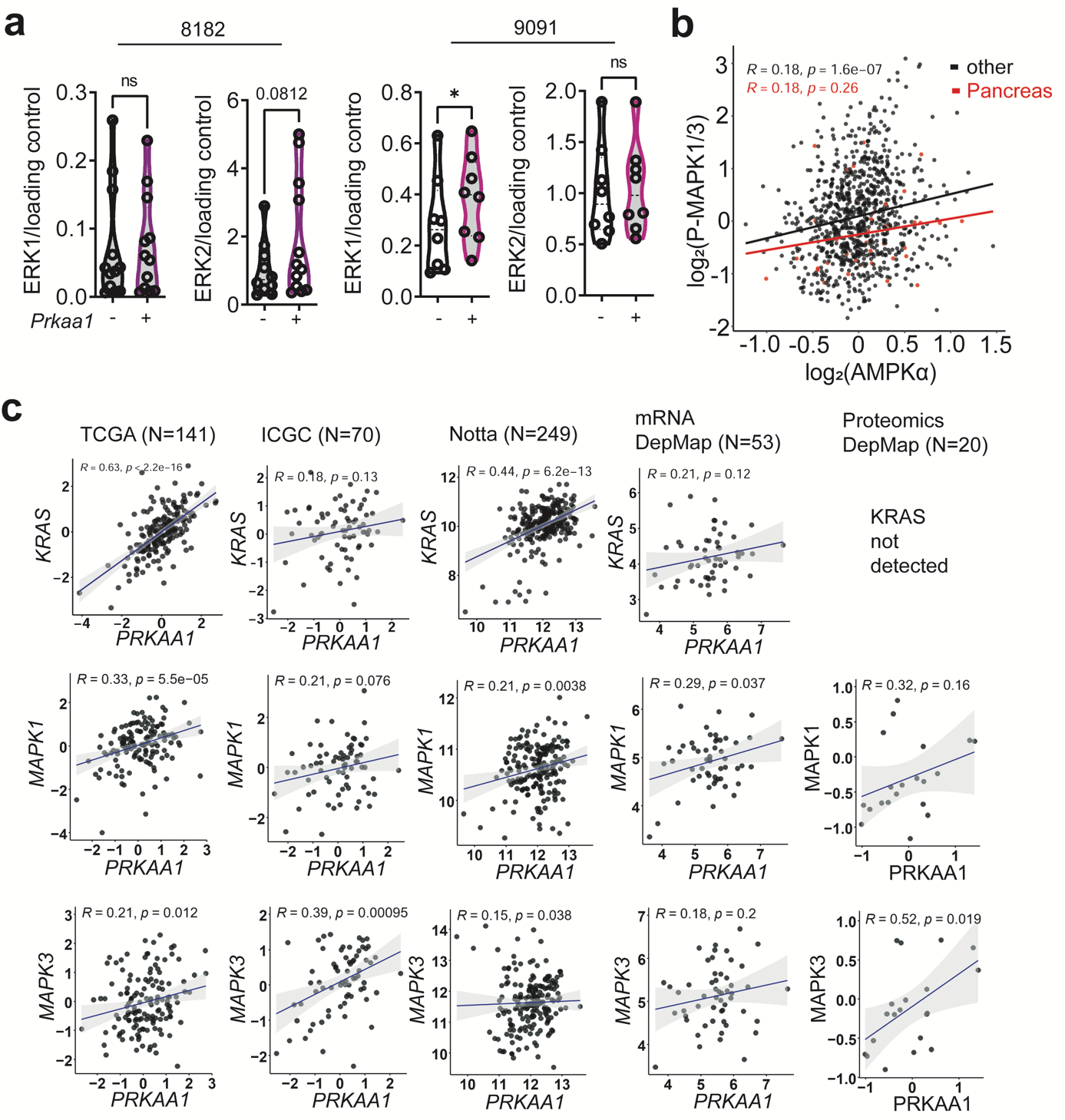


**Figure S5 | Prkaa1 correlates with canonical KRAS signaling. a,** Quantification of **Figure 2 h,**. **b,** Correlation of p-ERK at Threonine 202 and Tyrosine 204 (MAPK_pT202_Y204) and AMPKα (AMPKalpha) using Protein Array data accessed via <https://depmap.org/>. Pearson correlation coefficient (R) and p-value (p) for cancer cell lines of the pancreas is indicated in red and for other entities in black. **c,** Correlation of Prkaa1 with KRAS, ERK1 (MAPK3) and ERK2 (MAPK1) in TCGA, ICGC, Notta, RNA-Seq (<https://depmap.org/>) and proteomics (<https://depmap.org/>) data. TCGA, ICGC and proteomics data are shown as z-score, Notta and RNA-Seq data are shown as log_2_-transformed values. Pearson correlation coefficient (R) and p-value (p) are shown. ns: not significant, *: p<0.05.

**
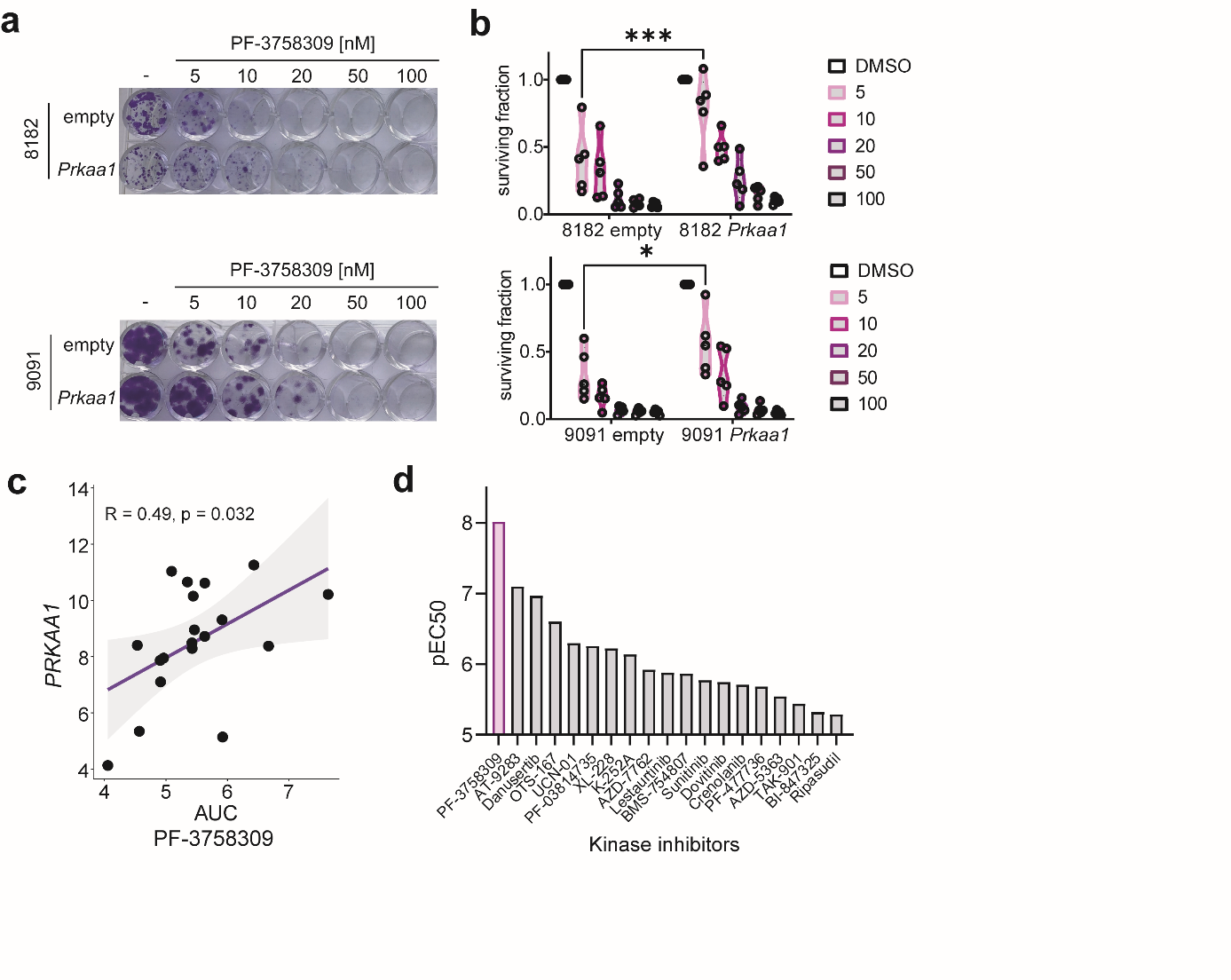
**

**Figure S6 | *Prkaa1* causes resistance towards PF-3758309. a,** Clonogenic assay of empty and *Prkaa1* overexpressing PDAC cells treated with different concentrations of PF-3758309 for 8 days. **b,** Quantification of **a,** On the y-axis, the surviving fraction of empty and Prkaa1 overexpressing cells treated with different concentrations of PF-3758309 [nM] after 8 days is depicted. Experiments were performed as five biological replicates. **c**, Correlation of PRKAA1 with CTD^2^ AUC of PF-3758309 (https://depmap.org/) and proteomic (https://depmap.org/) data. Pearson correlation coefficient (R) and p-value (p) are shown. **d,** Effective concentration 50 (EC_50_) of potential inhibitors for PRKAA1, depicted as log_10_(EC_50_M), were accessed via ProteomicsDB. *: p<0.05, ***: p<0.005.


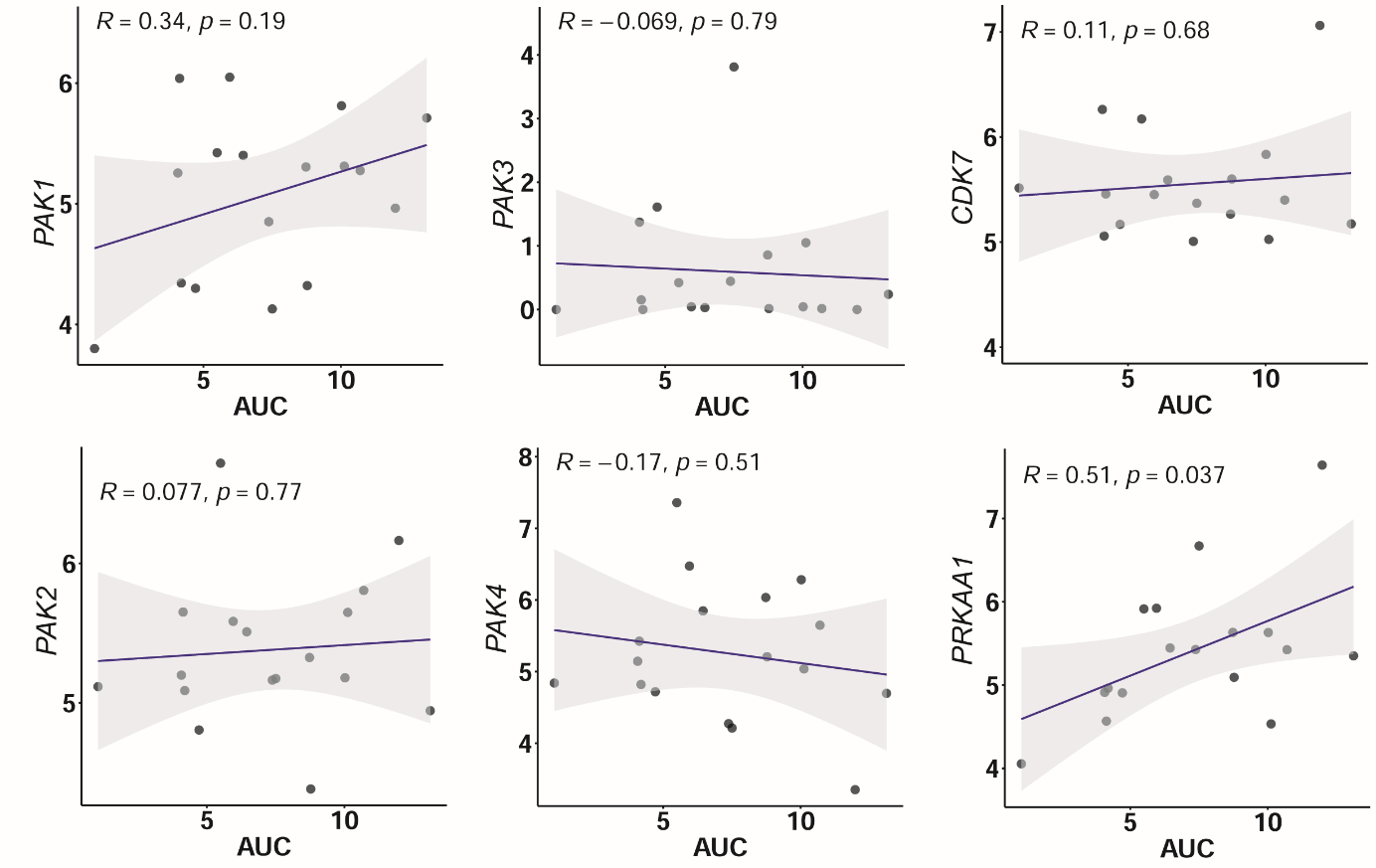


**Figure S7 | Correlation of AUC of PF-3758309 and potential targets beyond Prkaa1.** CTD^2^ AUC of PF-3758309 (https://depmap.org/) and mRNA expression (https://depmap.org/) data was used. Targets include *PAK1, PAK2, PAK3, PAK4* and *CDK7*. P: p-value, R: Pearson correlation coefficient.

**
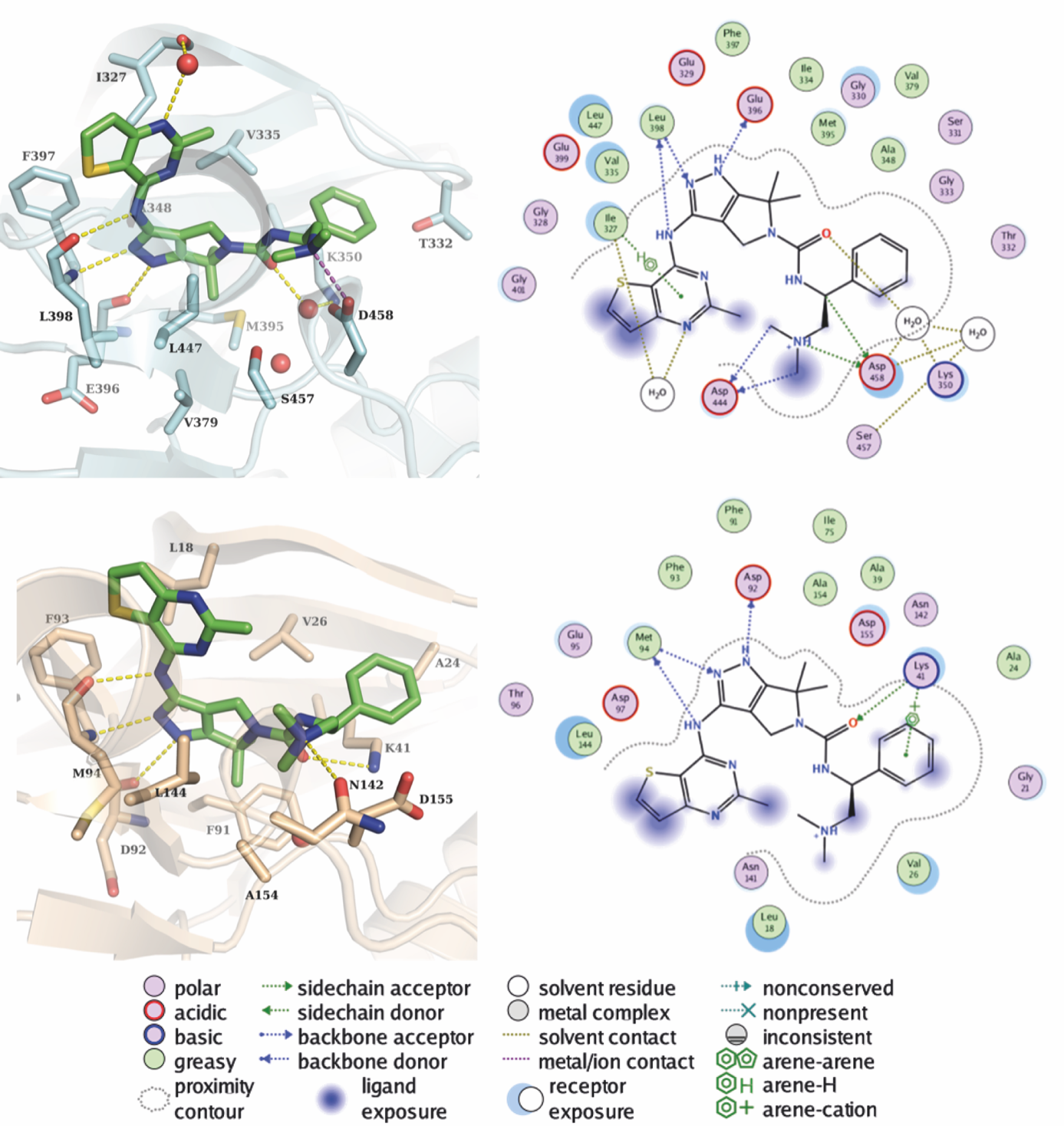
**

**Figure S8 |** Interaction of PAK4 (PDB ID 2XZ4) (upper panel) and CDK7 (PDB ID 7B5Q) (lower panel) with cocrystallized PF-3758309. Right: 2D plot of kinase-ligand interaction. Left: 3D representation of the binding of the inhibitor (colored green) to the ATP pocket. Hydrogen bonds are shown as yellow-colored dashed lines. Salt bridges are shown as magenta-colored dashed lines. Water molecules are displayed as red spheres.


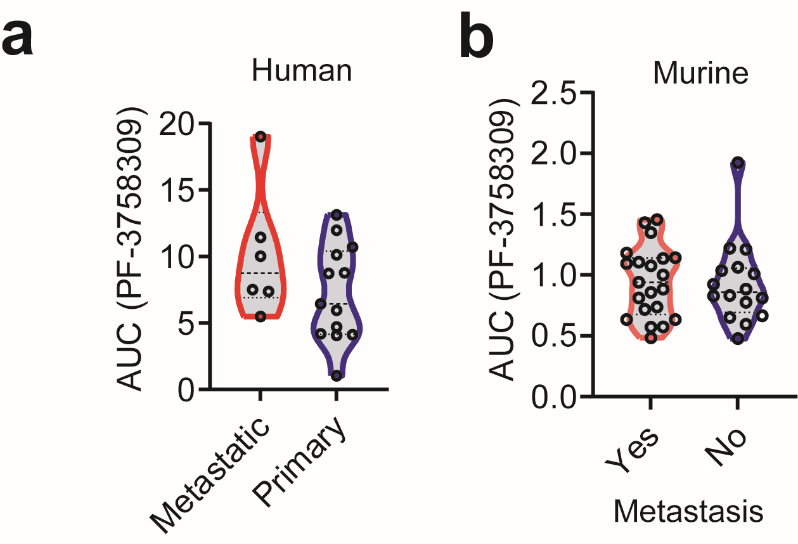


**Figure S9 | Response of PF-3758309 and metastasis.** **a,** AUC of human PDAC cell lines treated with PF-3758309 using the CTD^2^ screen data (https://depmap.org/) in cell lines derived from metastatic sites or primary tumors. **b,** AUC of murine PDAC cell lines from primary tumors stratified according to metastatic status upon sacrifice. Murine PDAC cells and annotations are derived from reference 38. Please note that all murine cell lines were isolated from pancreatic primary cancers.


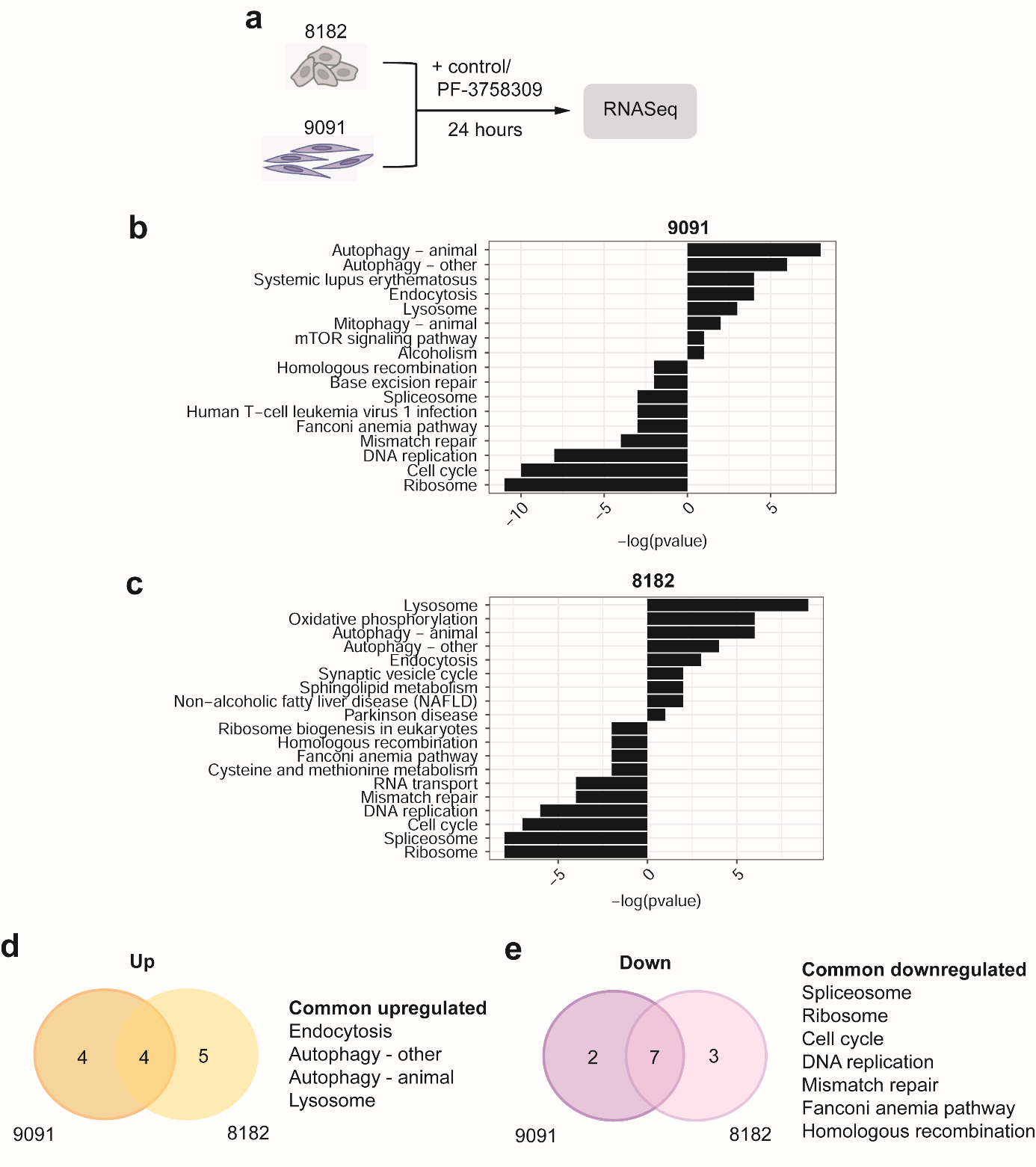


**Figure S10 | PF-3758309 controlled pathways. a,** 9091 and 8182 empty cells were treated for 24 hours with 25 nM PF-3758309. Genetrail 3.2 was used to investigate KEGG gene sets. Experiments were performed as three biological replicates. **b,** Top 10 up and downregulated signatures in 9091 empty cells. -log_10_(pvalue) is shown on the x-axis and signatures are shown on the y-axis. **c,** Top 10 up and downregulated signatures in 8182 empty cells. -log_10_(pvalue) is shown on the x-axis and signatures are shown on the y-axis.**d,** Venn diagram of top upregulated signatures in 9091 and 8182 empty cells. Common upregulated signatures are depicted on the right. **e,** Venn diagram of top downregulated signatures in 9091 and 8182 empty cells. Common downregulated signatures are depicted on the right.


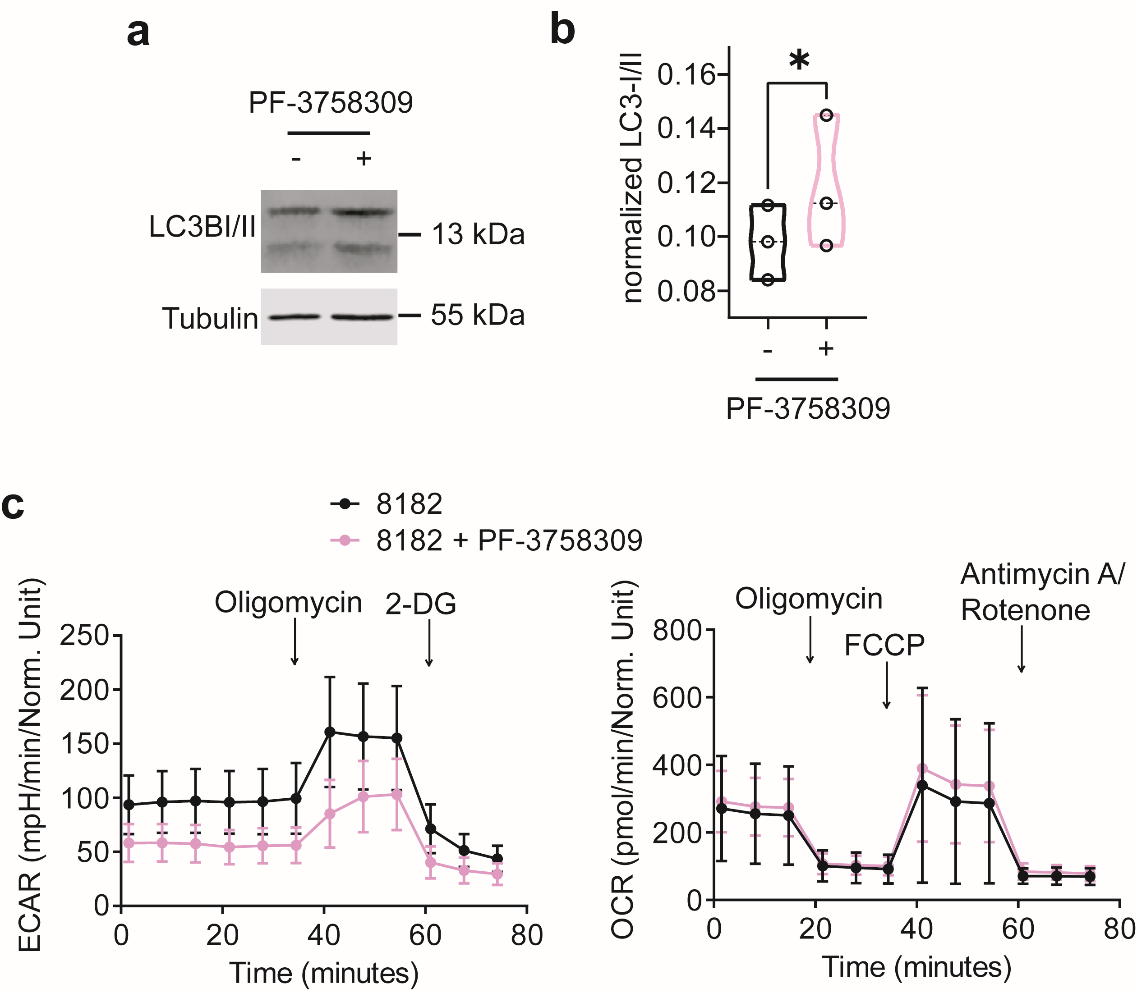


**Figure S11 | Metabolic alterations upon PF-3758309 treatment. a,** Western blot analysis of LC3BI/II upon PF-3758309 or control treatment in 8182 empty cells. Experiments were performed as biological triplicates. **b,** Quantification of **a,**. One-sided t-test was performed. **c,** Seahorse assay of 8182 empty cells upon 6 hours of 100 nM PF-3758309 or control treatment. Extracellular acidification rate (ECAR) and Oxygen consumption rate (OCR) were determined. Experiments were performed as five technical replicates and three biological replicates. *:p<0.05.


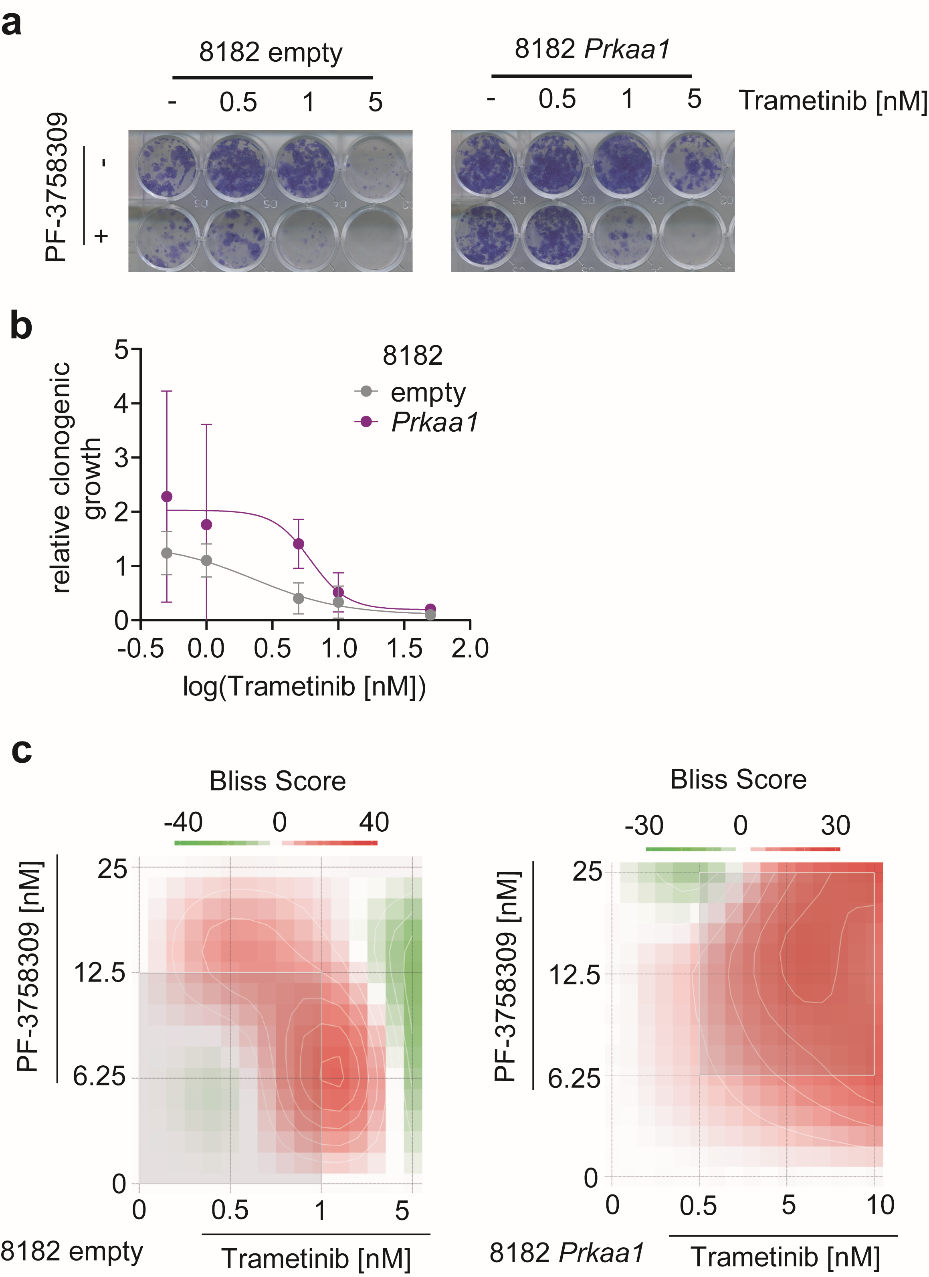


**Figure S12** **| *Prkaa1* is associated with resistance towards Trametinib. a,** Clonogenic assay of 8182 empty and *Prkaa1* overexpressing cells treated with different concentrations of Trametinib and PF-3758309 for 8 days. **b,** Quantification of **a,** for Trametinib**.** On the y-axis, the relative clonogenic growth of 8182 empty and *Prkaa1* overexpressing cells treated with different concentrations of Trametinib normalized to control treated cells is depicted. Experiments were performed as three biological replicates. **c,** Bliss synergy scores of **a,** for 8182 empty and *Prkaa1* overexpressing cells treated with indicated concentrations of Trametinib and PF-3758309 for 8 days. Experiments were performed as three biological replicates. Data was analyzed using https://synergyfinder.fimm.fi/.

**
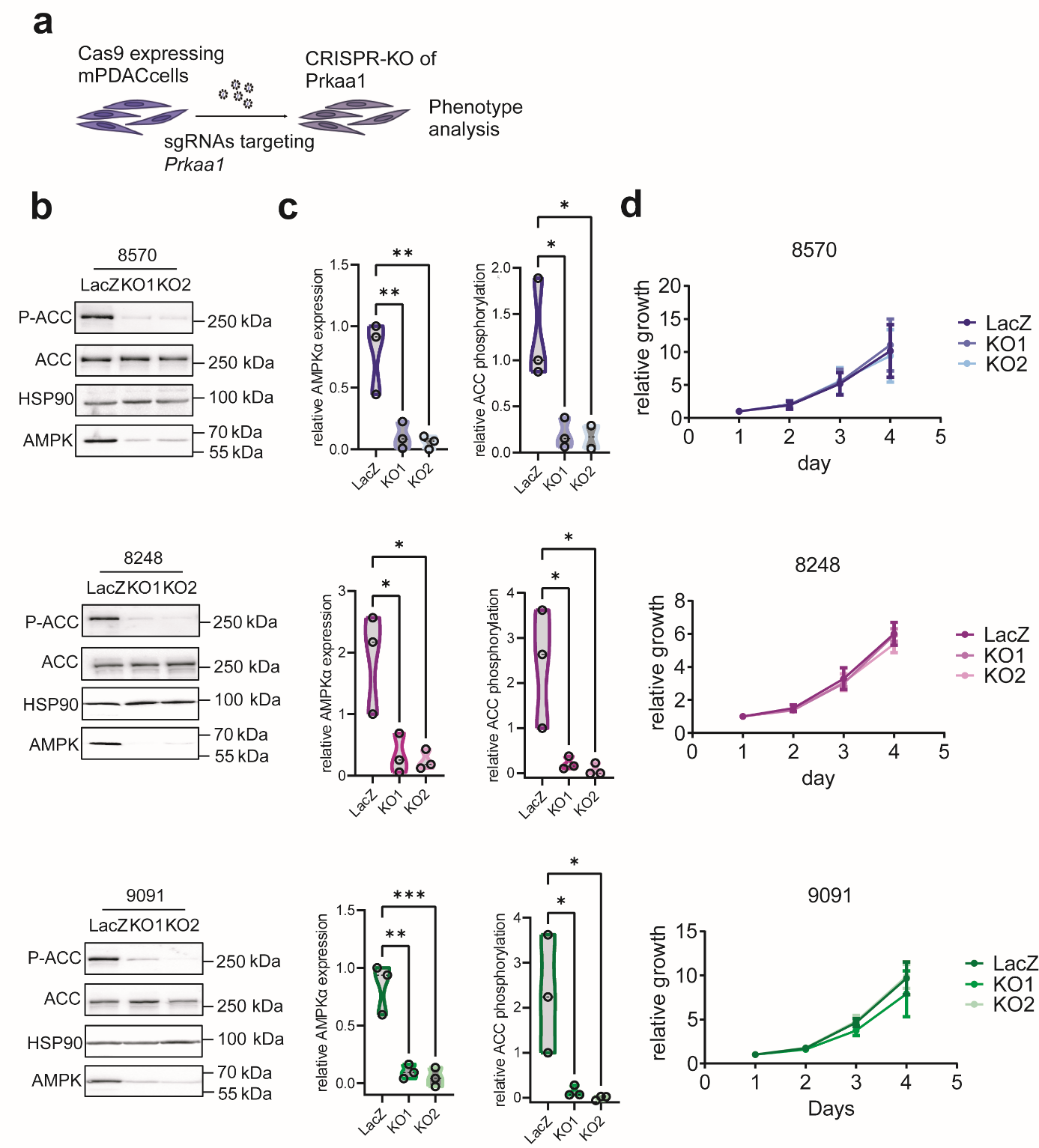
**

**Figure S13 | Characterization of the cellular *Prkaa1* knock-out phenotype. a,** Scheme of generation of *Prkaa1* KO cell lines. CAS9 expressing murine PDAC (mPDAC) cell lines were transduced with two different *Prkaa1*-targeting sgRNAs or a control sgRNA and subsequently their phenotype was analyzed. **b,** Western blots of AMPK pathway in control (LacZ) and Prkaa1 knockout (KO1, KO2) cells. The AMPK pathway was investigated using AMPKα, P-ACC, and ACC antibodies. Hsp90 was used as the loading control. **c,** Quantification of **b,**. For statistical analysis, one-way ANOVA with Tukey’s multiple comparisons was performed. **d,** Growth curves of control (LacZ) and *Prkaa1* knockout (KO1, KO2) cells. 1,000 cells were seeded in 96-well plates and viability was measured each day for four subsequent days. Relative growth to day one is plotted on the y-axis. The experiment was performed with three technical replicates and two (8248) or three (8570, 9091) biological replicates. ACC: Acetyl-CoA carboxylase, Hsp90: Heat shock protein 90, P-: Phosphorylation, ns: not significant, *: p<0.05, **: p<0.01, ***: p<0.005.

**
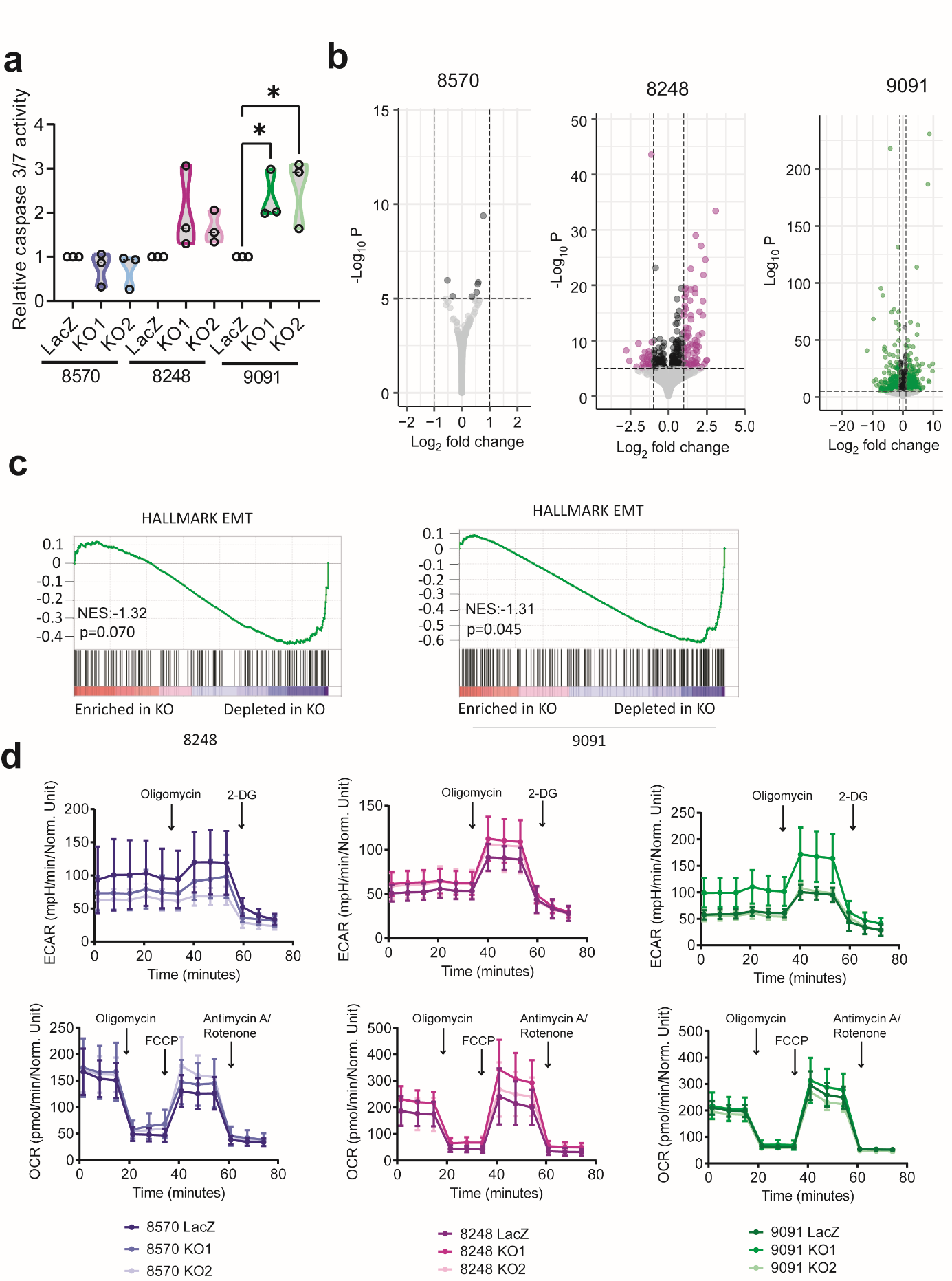
**

**Figure S14 | *Prkaa1* knock-out associated phenotypes. a,** Relative caspase 3/7 activity in LacZ vs. KO1 and KO2 cells. Caspase 3/7 activity was normalized to LacZ cells. Experiments were performed as two technical and three biological replicates. For statistical analysis, one-way ANOVA with Tukey’s multiple comparisons was performed. **b,** Volcano plot of differently expressed genes in LacZ vs. Prkaa1 KO cells determined by RNA-Seq. Log_2_ fold change is plotted on the x-axis and the log_10_(p-value) is plotted on the y-axis. mRNA of three (8570, 9091) or four (8248) biological replicates were used for processing. **c,** GSEA of RNA-Seq data in LacZ vs. KO cells using the EMT-HALLMARK gene set. Normalized enrichment scores (NES) and p-values (p) are shown. **d,** Seahorse assay of *Prkaa1* KO cell lines. Cell lines are color-coded. Upper: On the y-axis, the ECAR in mpH/min is shown. On the x-axis, the Time in minutes is given. Lower: On the y-axis, OCR in pmol/min is shown. On the x-axis, the Time in minutes is given. Experiments were performed as two biological replicates. ECAR: Extracellular acidification rate, OCR: Oxygen consumption rate,*: p<0.05.

**
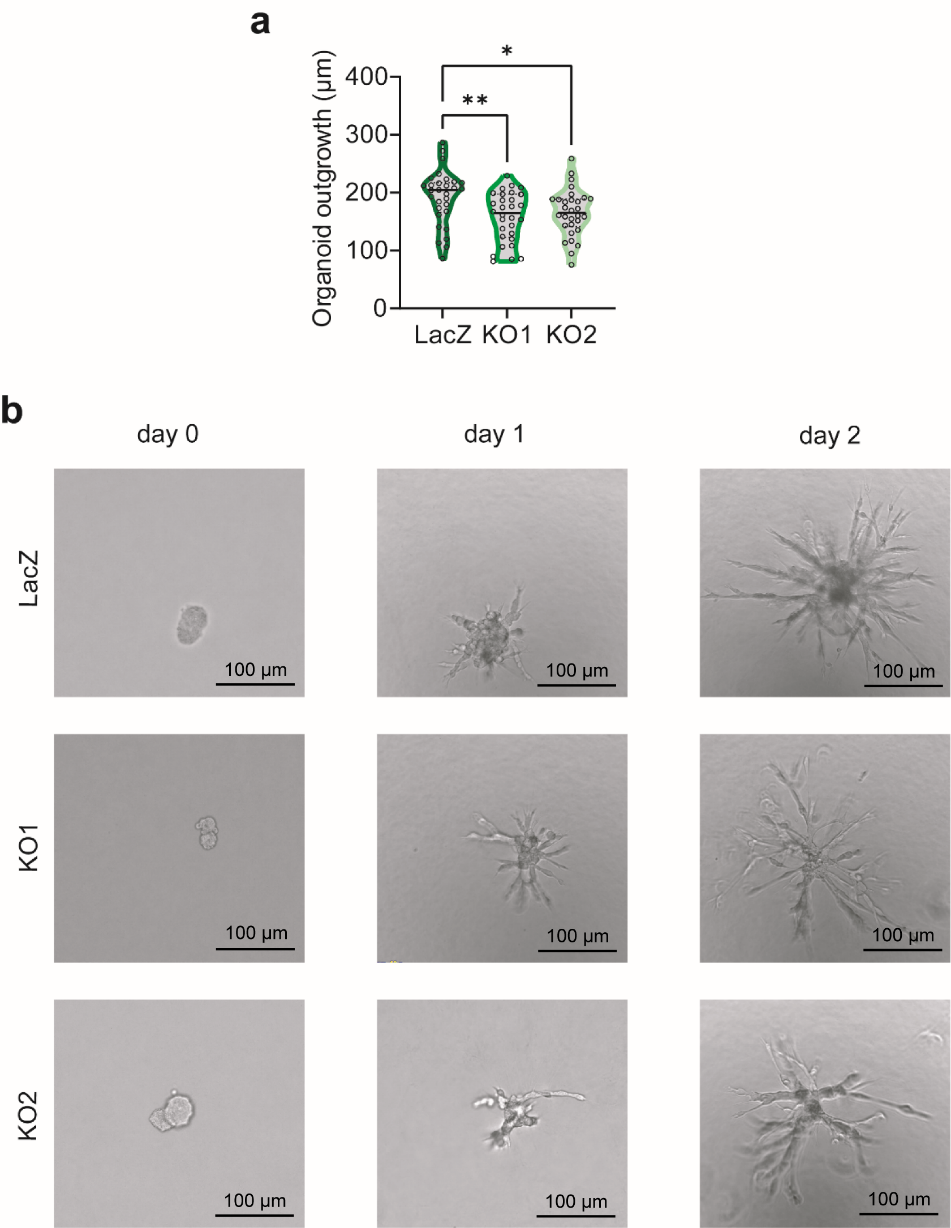
**

**Figure S15 | Organoid branching of *Prkaa1* knock-out cells.** 9091 LacZ control cells and respective *Prkaa1* KO1 and *Prkaa1* KO2 cells were used. Microscopic pictures were taken after day 0, day 1 and day 2 and organoid outgrowth was determined relative to organoid growth on day 0. **a,** Quantification of organoid outgrowth in µm. **b,** Representative microscopic pictures of 9091 LacZ control cells and respective *Prkaa1* KO1 and *Prkaa1* KO2 cells after day 0, day 1 and day 2 upon matrix transfer.

**
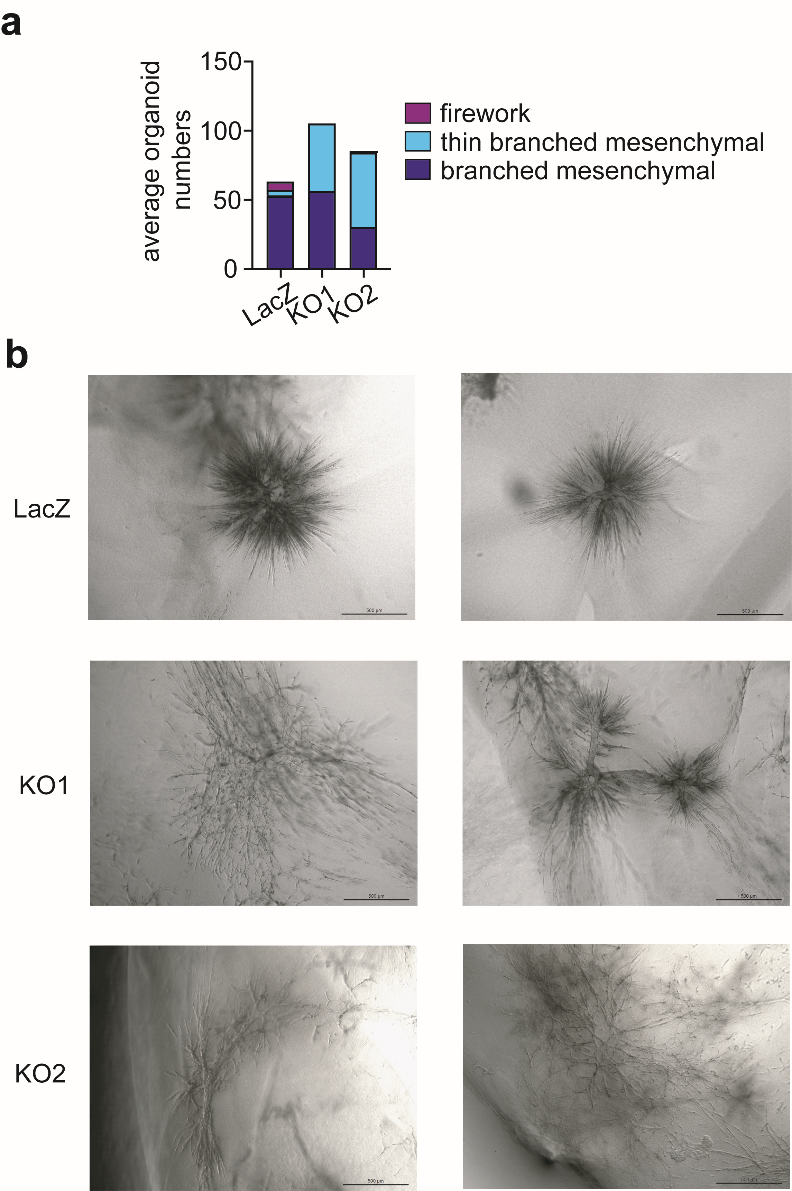
**

**Figure S16 | Organoid growth phenotype of *Prkaa1* knock-out cells.** 9091 LacZ control cells and respective *Prkaa1* KO1 and *Prkaa1* KO2 cells were used. Microscopic pictures were taken after 13 days and organoid growth phenotype was determined. **a,** Quantification of organoids and phenotype of respective organoids. **b,** Representative microscopic pictures of 9091 LacZ control cells and respective *Prkaa1* KO1 and *Prkaa1* KO2 cells.

**
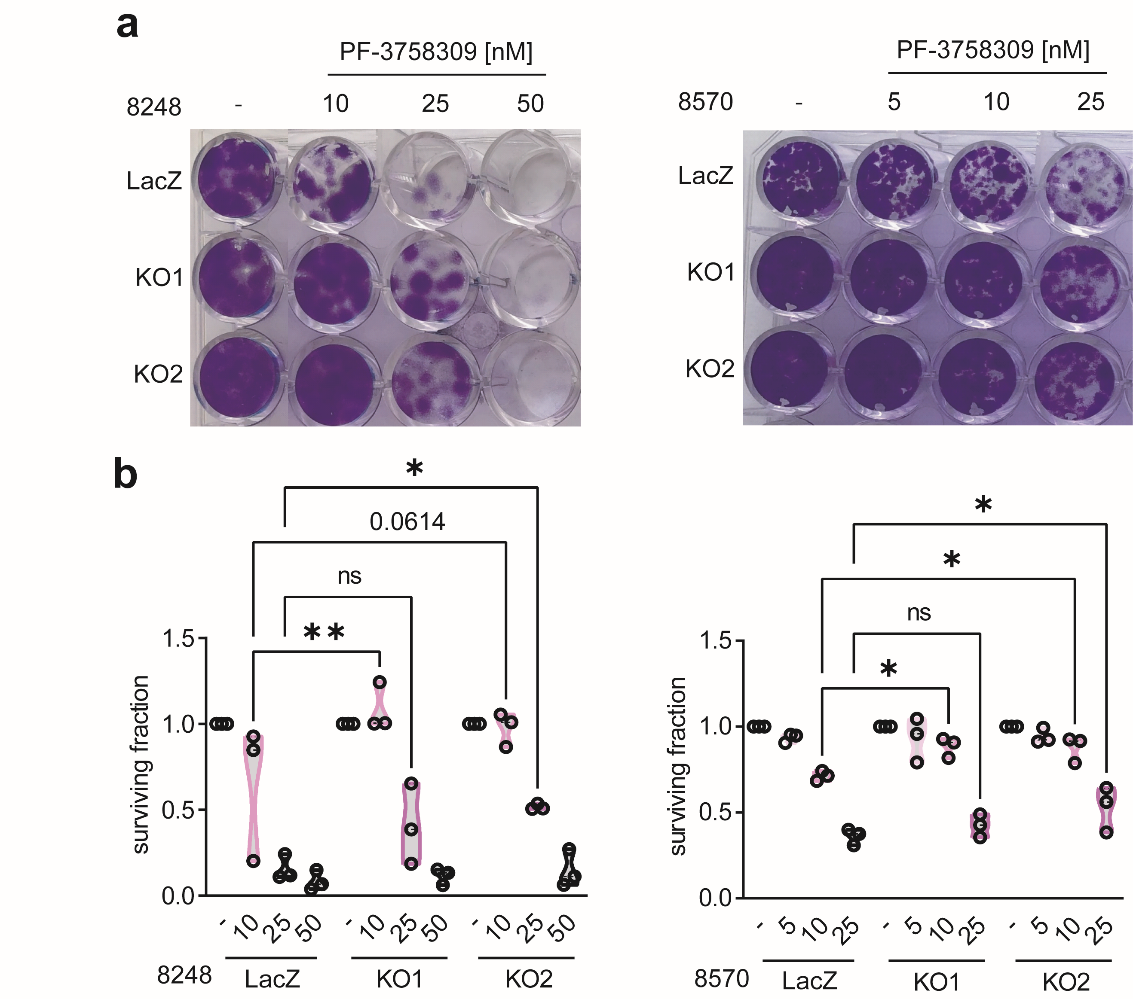
**

**Figure S17 | AMPK inhibition might contribute to the PF-3758309 response. a,** Clonogenic assay of 8248 and 8570 LacZ control cells and their respective *Prkaa1* KO1, and *Prkaa1* KO2 cells treated with indicated concentrations of PF-3758309 [nM] for 8 days. **b,** Quantification of **a,**. Surviving fraction is shown on y-axis. For statistical analysis, one-way ANOVA with Bonferroni’s multiple comparisons was performed.

**
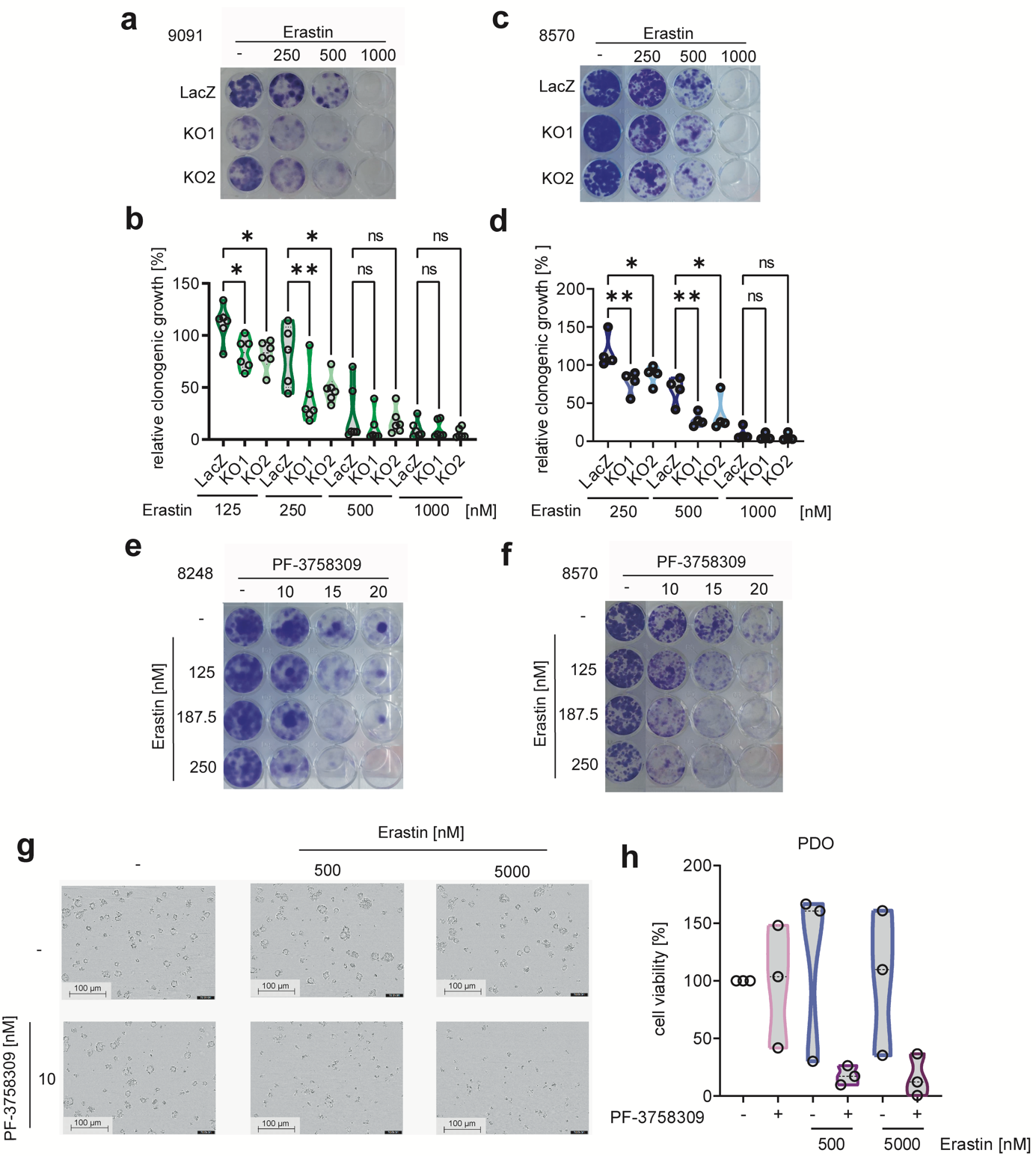
**

**Figure S18 | Erastin responsiveness is controlled by AMPK. a,** Clonogenic assay of 9091 LacZ control cells and respective *Prkaa1* KO1, and *Prkaa1* KO2 cells treated with indicated concentrations of Erastin [nM] for 8 days. Experiments were performed as six biological replicates. **b,** Quantification of **a,**. **c,** Clonogenic assay of 8570 LacZ control cells and respective *Prkaa1* KO1, and *Prkaa1* KO2 cells treated with indicated concentrations of Erastin [nM] for 8 days. Experiments were performed as six biological replicates. **d,** Quantification of **c,**. **e,** Clonogenic assay of 8570 LacZ control cells treated with indicated concentrations of Erastin [nM] and PF-3758309 [nM] for 8 days. **f,** Clonogenic assay of 8248 LacZ control cells treated with indicated concentrations of Erastin [nM] and PF-3758309 [nM] for 8 days. **g,** Live cell imaging of a human PDAC PDO line treated with PF-3758309 [nM] and/or Erastin [nM] as indicated. **h,** A PDAC PDO line was treated as indicated and viability was measured 72 hours after the treatment using ATP as a surrogate for the viability. ns: not significant, *: p<0.05, **: p<0.01.

*
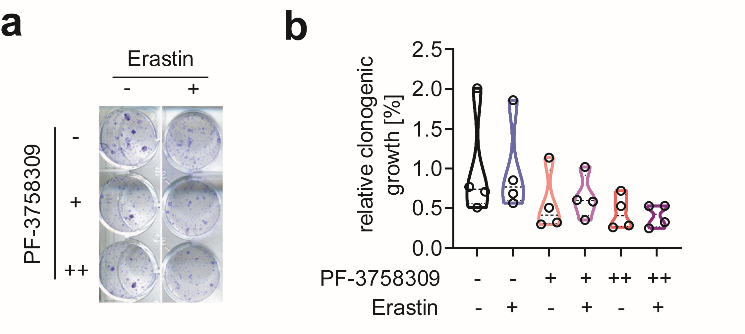
*

**Figure S19 | PF-3758309 response in HaCaT cells.** Clonogenic assay of HaCaT cells with PF-3758309 and Erastin. Cells were treated with PF-3758309 (+: 6.25 nM, ++: 12.5 nM) and 1 µM Erastin for 7 days. **a,** Picture of clonogenic assay. **b,** Quantification shown as relative clonogenic growth normalized to control. Experiments were performed as four biological replicates.

**
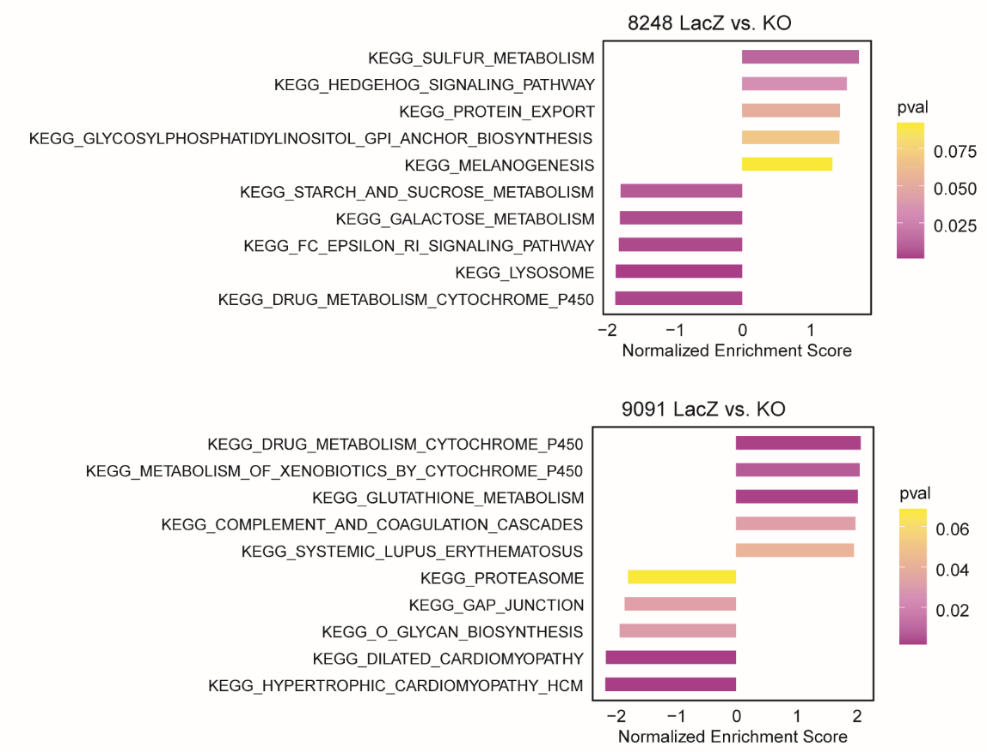
**

**Figure S20 | AMPK is connected to detoxifying pathways.** GSEA of RNA-Seq data of LacZ vs. KO cells using the KEGG gene set database. Depicted are the top 5 enriched and depleted KEGG gene sets. Normalized enrichment scores are shown on the x-axis and p-values (pval) are color-coded.


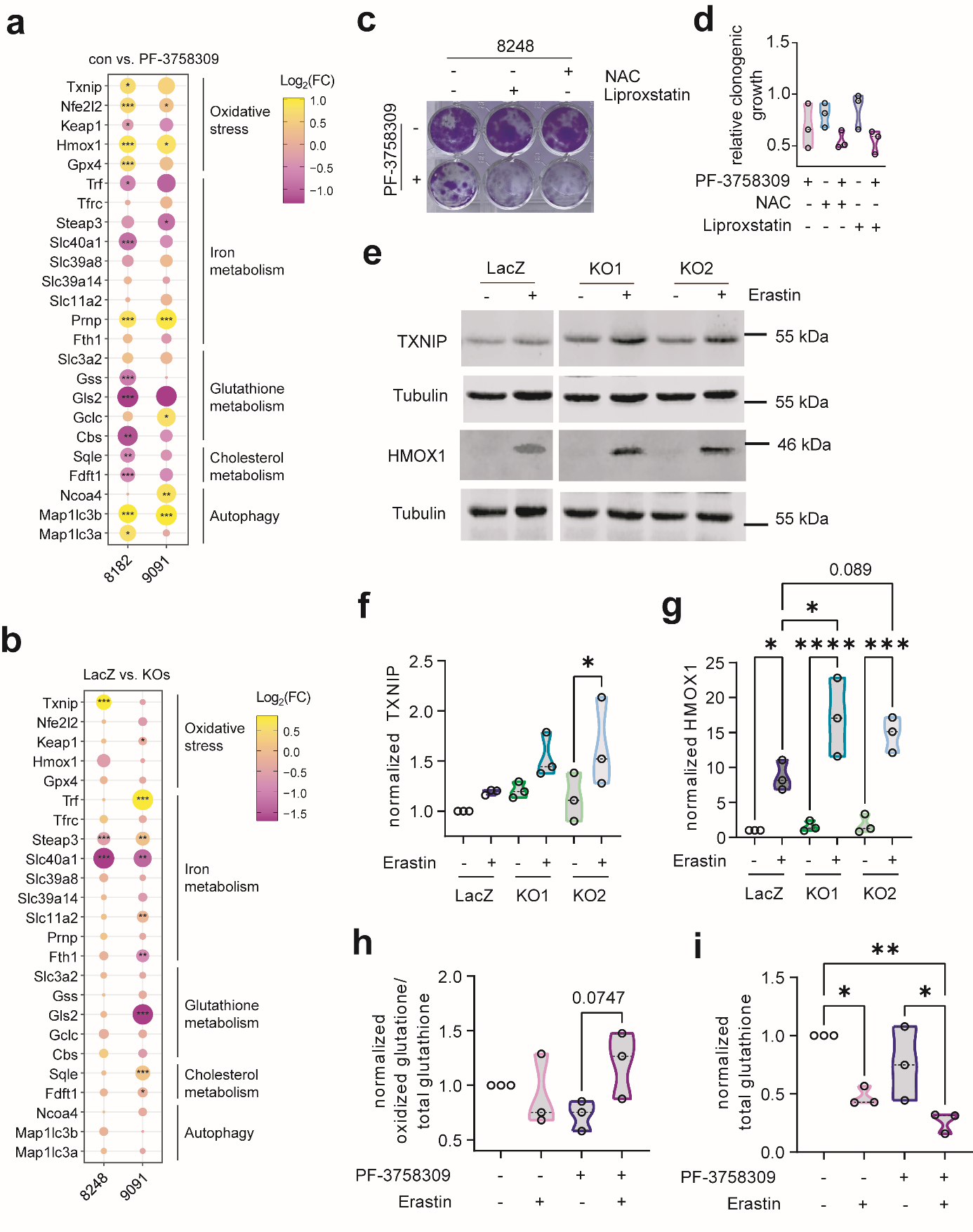


**Figure S21 |** **AMPK connected** **ferroptosis markers.** **a,** mRNA expression of ferroptosis-associated genes using RNASeq. Log_2_(FC) of mRNAs is color-coded. Pathways of genes are indicated. 8182 and 9091 cells were treated with PF-3758309 for 24 hours. PF-3758309 treatment compared to control are shown. **b,** mRNA expression of ferroptosis-associated genes using RNASeq. Log_2_(FC) of mRNAs is color-coded. Pathways of genes are indicated. KOs compared to LacZ control cells are shown. 8248 and 9091 LacZ control cells and respective *Prkaa1* KO1 and *Prkaa1* KO2 cells were used. **c,** Clonogenic assay and **d,** quantification of 8248 LacZ control cells treated with 6.25 nM PF-3758309, 0.5 µM Liproxstatin and 2 µM NAC for 7 days. Experiments were performed as three biological replicates. A representative clonogenic assay is shown. Cell growth was normalized to control and is plotted as relative clonogenic growth. NAC: N-Acetylcysteine. **e,** 9091 LacZ control cells and respective *Prkaa1* KO1 and *Prkaa1* KO2 cells were treated with 500 nM Erastin for 24 hours and levels of TXNIP and HMOX1 were investigated by western blot. Experiments were performed as biological triplicates **f,** Quantification of TXNIP. TXNIP was normalized to loading control: Tubulin. **g,** Quantification of HMOX1. HMOX1 was normalized to loading control: Tubulin. **h,** Glutathione assay of 9091 LacZ control cells treated with 250 nM Erastin and/or 15 nM PF-3758309 for 48 hours. Experiments were performed as biological triplicates. Glutathione levels were normalized to cell viability. Ratio of oxidized glutathione to total glutathione. Ratios were normalized to control treatment. **i,** Glutathione assay of 9091 LacZ control cells treated with 250 nM Erastin and/or 15 nM PF-3758309 for 48 hours. Experiments were performed as biological triplicates. Glutathione levels were normalized to cell viability. Total glutathione was normalized to control treatment. *:p-value<0.05, **:p-value<0.01, ****:p-value<0.0001.
